# Genomic differentiation in Pacific cod using Pool‐Seq

**DOI:** 10.1111/eva.13488

**Published:** 2022-10-13

**Authors:** Ingrid Spies, Carolyn Tarpey, Trond Kristiansen, Mary Fisher, Sean Rohan, Lorenz Hauser

**Affiliations:** ^1^ Resource Ecology and Fisheries Management Division Alaska Fisheries Science Center Seattle Washington USA; ^2^ School of Aquatic and Fishery Sciences University of Washington Seattle Washington USA; ^3^ Farallon Institute, Inc. Petaluma California USA; ^4^ Resource Assessment and Conservation Engineering Division Alaska Fisheries Science Center Seattle Washington USA

**Keywords:** genomic differentiation, genomics, local adaptation, Pacific cod, Pool‐Seq, selection, selection–migration balance

## Abstract

Patterns of genetic differentiation across the genome can provide insight into selective forces driving adaptation. We used pooled whole genome sequencing, gene annotation, and environmental covariates to evaluate patterns of genomic differentiation and to investigate mechanisms responsible for divergence among proximate Pacific cod (*Gadus macrocephalus*) populations from the Bering Sea and Aleutian Islands and more distant Washington Coast cod. Samples were taken from eight spawning locations, three of which were replicated to estimate consistency in allele frequency estimation. A kernel smoothing moving weighted average of relative divergence (*F*_ST_) identified 11 genomic islands of differentiation between the Aleutian Islands and Bering Sea samples. In some islands of differentiation, there was also elevated absolute divergence (*d*_XY_) and evidence for selection, despite proximity and potential for gene flow. Similar levels of absolute divergence (*d*_XY_) but roughly double the relative divergence (*F*_ST_) were observed between the distant Bering Sea and Washington Coast samples. Islands of differentiation were much smaller than the four large inversions among Atlantic cod ecotypes. Islands of differentiation between the Bering Sea and Aleutian Island were associated with SNPs from five vision system genes, which can be associated with feeding, predator avoidance, orientation, and socialization. We hypothesize that islands of differentiation between Pacific cod from the Bering Sea and Aleutian Islands provide evidence for adaptive differentiation despite gene flow in this commercially important marine species.

## 1 INTRODUCTION

Comparison among genomes of allopatric and parapatric populations may show heterogeneous levels of differentiation, depending on the level of divergence (Nosil et al., 2009), and may provide insight into the evolution and maintenance of adaptive divergence despite gene flow (Graham et al., 2018; Monnahan et al., 2015). Studies of nonmodel species indicate that selection at specific genome regions may allow local adaption even in the presence of gene flow (Barth et al., 2017; Dennenmoser et al., 2017). Regions of significantly elevated differentiation have been referred to as ‘genomic islands of differentiation’ (Wolf & Ellegren, 2017) or ‘genomic islands of speciation’ (Turner et al., 2005). We use the phrase ‘genomic islands of differentiation’ because here we focus on local adaptation rather than speciation and these regions are not necessarily associated with speciation.

Natural selection in different environments may influence the emergence and persistence of islands of differentiation, resulting from a complex range of mechanisms (Yeaman, 2013). Islands of differentiation can arise from chromosomal rearrangements, selection with hitchhiking, and background selection. Low recombination in regions of genomic rearrangements may facilitate the evolution of islands of differentiation, because genetic linkage reduces recombination of adaptive combinations of alleles (Via, 2012; Yeaman, 2013). Chromosomal rearrangements, or inversions, restrict recombination in heterokaryotypes and can be adaptive or neutral (Lotterhos, 2019; Noor & Bennett, 2009). Background selection can result in increased levels of differentiation due to selection against deleterious alleles at linked loci (Charlesworth et al., 1993). Selection with hitchhiking occurs when regions of the genome subject to strong selection promote secondary changes in gene frequencies in closely linked regions (Smith & Haigh, 1974; Wolf & Ellegren, 2017).

The capacity for local adaptation rests on a balance between the strength of selection and gene flow, or migration–selection balance (Graham et al., 2018). During the emergence of local adaptation among connected populations, the majority of the genome is thought to be subject to gene flow and few divergent clusters are expected (Nosil et al., 2009; Via, 2012). Stronger selection is thought to cause islands of differentiation to increase in size (Graham et al., 2018; Nosil et al., 2009; Yeaman & Whitlock, 2011). Background selection against strongly deleterious mutations can also lead to nonneutral distortions in the allele frequency spectrum (Charlesworth et al., 1993; Cvijović et al., 2018; Good et al., 2014). In a region in which the deleterious mutation rate is elevated, there will be an excess of rare alleles because selection cannot act instantaneously (Cvijović et al., 2018; Good et al., 2014). As such, distinguishing background selection from positive selection and selection with hitchhiking might not be possible because they may both lead to an excess of rare alleles in the site frequency spectrum (Cvijović et al., 2018). Nevertheless, a comparison of relative (*F*_ST_) and absolute (*d*_XY_) differentiation between populations with Tajima's D and nucleotide diversity within populations may provide insights into the relative importance of selection and migration in shaping patterns of genome variability.

Selection in connected populations is particularly relevant in marine species supporting commercial fisheries to understand the biological basis behind established management units. Genetic population structure has been documented among parapatric groups of Pacific cod that spawn in ecologically and biophysically different regions across their range (Drinan et al., 2018; Spies, 2012; Spies et al., 2020). In Alaska, Pacific cod are managed in three management units corresponding to the Gulf of Alaska, Aleutian Islands, and eastern Bering Sea. A general pattern of isolation by distance among spawning groups of Pacific cod (Cunningham et al., 2009; Drinan et al., 2018; Spies, 2012) is punctuated by locally pronounced genetic differentiation between the Aleutian Islands and eastern Bering Sea (Spies, 2012) and between the western and eastern Gulf of Alaska (Drinan et al., 2018; Spies et al., 2021). However, divergence across the genome between Aleutian Islands and eastern Bering Sea cod has not been examined. Such data may further elucidate levels of connectivity as well as mechanisms of local adaptation in these areas, which support the largest cod fishery in the United States, with an average annual gross value of $379 million between 2016 and 2020 (Fissel et al., 2020).

Examination of adaptive divergence in Pacific cod is also merited given differences observed in physical environment and prey of cod in these two regions. The Bering Sea contains a prominent and broad (500 km) continental shelf adjacent to a deep‐sea basin that stretches from the Alaska Peninsula to the Bering Strait. In contrast, the Aleutian Islands consist of a narrow steep shelf formed by an 1800 km long chain of volcanic mountaintops (Logerwell et al., 2005). While Pacific cod occurs in the western and eastern Bering Sea (O'Leary et al., 2022), we focus on Pacific cod in the United States management region of eastern Bering Sea (Stevenson & Lauth, 2019). The eastern Bering Sea (EBS) continental shelf consists of three depth regions: the inner (0–50 m depth), middle (50–100 m), and outer domain (100–180 m; Stabeno et al., 2012). The extent of sea ice in the Bering Sea during winter varies interannually, but has declined in recent years (Grebmeier et al., 2006; Stevenson & Lauth, 2019). The timing of the maximum ice extent and its retreat affects the species composition across a range of taxa from the zooplankton community to higher trophic level predators (Stevenson & Lauth, 2019). The physical environment of the Aleutian Islands is dynamic; islands are separated by passes that allow the transfer of water from the North Pacific Ocean northward and the water quality and species distributions change along the chain in response to shifting currents and passes (Hunt Jr & Stabeno, 2005; Logerwell et al., 2005). For example, there is an abrupt shift in the physical environment and species composition at Samalga Pass (169° W), an area that divides warmer, nutrient‐poor coastal waters of the Alaska Coastal Current to the east and the colder, nutrient‐rich oceanic Alaskan Stream to the west (Hunt Jr & Stabeno, 2005).

These distinct physical environments, and the resulting shifts in ecological communities, drive diet differences between Pacific cod in the Aleutian Islands and EBS. Walleye pollock (*Gadus chalcogrammus*) are a major diet component of Pacific cod in the EBS (26%), but in the Aleutians, Atka mackerel (*Pleurogrammus monopterygius*) and sculpins are the predominant fish prey (15% each), while pollock comprise only 5% (Aydin et al., 2007). Snow and tanner crab (*Chionoecetes opilio* and *C*. *bairdi*) make up 9% of cod diets in the EBS, but less than 3% in the Aleutian Islands. In contrast, squids comprise over 6% of cod diets in the AI but are a negligible proportion of diets in the EBS. Myctophids (family Myctophidae) are also found only in cod diets in the Aleutian Islands, but not in the EBS even though they are present in the diets of other EBS fish species (Aydin et al., 2007; Lang & Livingston, 1996).

Lessons from the congeneric Atlantic cod (*Gadus morhua*) may help inform the genomics of Pacific cod; in Atlantic cod, several ecotypes are known, including Northeast Arctic cod (NEAC) and the Norwegian coastal cod (NCC). The genomic basis of these ecotypes appears to be related to inversions on linkage groups 1, 2, 7, and 12 (Árnason & Halldórsdóttir, 2019; Barth et al., 2017; Hemmer‐Hansen et al., 2013). Genes within these inversions appear to be responsible for maintaining selective differences related to habitats and life history (Barth et al., 2017; Kirubakaran et al., 2016). It is currently unknown if such inversions also drive population structure in Pacific cod.

Some knowledge of the demographic history of Pacific cod is valuable for interpreting genomic differences. Pacific cod are believed to have originated from an Atlantic cod ancestor that moved into the Pacific Ocean 3.8 million years ago (Árnason & Halldórsdóttir, 2019). During Pleistocene glaciations, water levels fluctuated and the Bering Sea shelf was exposed during several time periods, forming the Bering land bridge as recently as 30,000–18,000 years ago (Elias et al., 1996). Glaciers extended from the Alaska Peninsula to the Aleutian chain through the southern extent of Alaska to northwestern Canada southward to Puget Sound (Batchelor et al., 2019), during which time cod likely migrated southward. Cod recolonized the North Pacific as glaciers receded, as recently as 14–15 kyr before present (Mann & Hamilton, 1995; Menounos et al., 2009). Analysis of the putative zona pellucida gene is consistent with this scenario, as the present‐day southern populations are more closely related to Atlantic cod and more northerly populations are more derived at this locus (Spies et al., 2021).

The aim of this study was to compare genomic patterns of differentiation among proximate versus distant Pacific cod populations from ecologically distinct regions and evaluate regions of the genome that may be subject to local adaptation. We applied Pool‐Seq to Pacific cod from the ecologically different Aleutian Islands and Bering Sea among which gene flow may occur, and to allopatric groups (EBS and Washington Coast), between which migration has not been observed despite thousands of tagging records (Bryan et al., 2021; Rand et al., 2014; Shimada & Kimura, 1994). These parapatric and allopatric groups were selected to provide examples across several levels of population divergence (Stankowski & Ravinet, 2021; Wolf & Ellegren, 2017). At the time this study was initiated, individual‐based whole genome sequencing was not a cost‐effective option. The power to identify departures from panmixia in marine fish with large effective population sizes increases with the number of genetic markers and individuals (Kalinowski, 2005; Vendrami et al., 2017; Waples & Gaggiotti, 2006). Therefore, we selected Pool‐Seq, which offers the capacity to sequence the genome more thoroughly than sequence capture (Harvey et al., 2016) or reduced representation sequencing (e.g., RAD‐seq, Andrews et al., 2016), and at a lower cost than individual‐based sequencing. Pool‐Seq also provides the combined contributions of individuals in the pool and has been shown to provide robust and reliable allele frequencies (Anand et al., 2016). The disadvantages to Pool‐Seq stem from the need to calculate allele frequencies over all pooled samples, which masks individual genotypes and precludes its utility for tests of linkage disequilibrium or population assignment (Dorant et al., 2019). In addition, unequal contributions of individuals in the pool can increase the error of allele frequency estimates (Rode et al., 2018). Nonetheless, Pool‐Seq has been used to successfully examine adaptive divergence and genetic population structure in a range of species (Dennenmoser et al., 2017; Dorant et al., 2019; Han et al., 2020; Kurland et al., 2019). This study was also well suited to Pool‐Seq because previous studies have defined genetic population structure and spawning units, and all cod were presumed to be in or near spawning stage (Cunningham et al., 2009; Spies et al., 2020).

A primary hypothesis was that genomic divergence between eastern Bering Sea and Aleutian Islands cod would manifest as a limited number of highly differentiated clusters if selection were sufficiently high, because gene flow is thought to be limited (Spies, 2012; Spies & Punt, 2015; Yeaman & Whitlock, 2011). We also expected that temperature would play a role in local adaptation, as winter bottom temperatures in the Aleutian Islands are roughly 2°C warmer in the winter than the Bering Sea, and cod are adapted to a relatively narrow temperature range at all life stages (Barbeaux et al., 2020; Hurst et al., 2010; Laurel et al., 2008). For example, in the Gulf of Alaska, Pacific cod has suffered recent declines in abundance due to the impact of marine heatwaves, and increased ocean temperatures are predicted under climate change (Barbeaux et al., 2020; Capotondi et al., 2012). Specifically, we hypothesized that (1) genetic differentiation between parapatric groups which may be subject to gene flow would vary across their genomes, while differentiation would be both more pronounced and more widespread across the genomes of allopatric groups, (2) regions of elevated divergence may provide clues to the selective mechanisms responsible for divergence among the parapatric Aleutian Islands and Bering Sea, and (3) genes underlying local adaptation of parapatric groups may be correlated with the environmental factors to which adaptation occurred, with temperature most likely to play a role. To address the first two hypotheses, we examined the parapatric and allopatric sets of samples for *F*_ST_ outlier regions and calculated nucleotide diversity **(** *π*), *d*_XY_, and Tajima's D to inform the level of gene flow and potential mechanisms shaping divergent regions. To address the final hypothesis, we identified four environmental covariates considered to impact Pacific cod: salinity, bottom temperature, surface chlorophyll, and current velocity, and quantified correlations between environmental covariates and allele frequencies. In addition, we screened for annotated genes within islands of differentiation among the Aleutian Islands and Bering Sea and measured light transmission to the seafloor in the Bering Sea and Aleutian Islands through bottom optical depth.

## 2 METHODS

### 2.1 Sample preparation and sequencing

**FIGURE 1:**
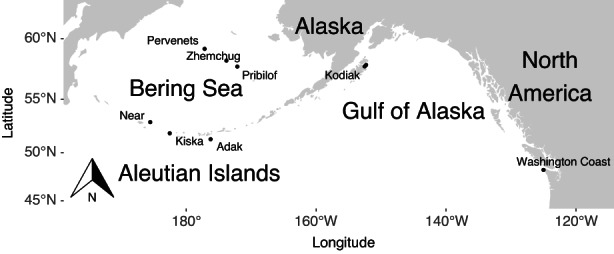
Spawning locations included in this study were named after nearby landmarks. There were three collections from the Bering Sea (Pervenets, Zhemchug, and Pribilof), three from the Aleutian Islands (Near Island, Kiska Island, and Adak Island), as well as a collection from Kodiak Island and the Washington Coast.

We analyzed Pacific cod fin clips from eight known spawning sites from the Aleutian Islands, EBS, Gulf of Alaska, and Washington Coast collected during February–May between 2003 and 2017 (Table 1, Figure 1). Studies of genetic stock structure in Pacific cod are typically limited to spawning fish because populations may intermingle outside the spawning season (Cunningham et al., 2009; Drinan et al., 2018; Rand et al., 2014). While Pacific cod typically spawn between January and April (Neidetcher et al., 2014), the Zhemchug sample, taken May 9, 2017, was considered close enough to that time window to be considered a late spawning sample. Individual gonadal condition was not recorded, but all samples were from adult cod that were taken from known spawning areas during spawning season and likely to be reproductively mature and in spawning condition. DNA from tissues stored in 95% ethanol from 88 to 96 individuals from each unique collection location and year (Table 1) were extracted using the DNeasy 96‐well Blood & Tissue Kits (Qiagen Inc.). The extracted DNA was quantified using Quant‐iT PicoGreen for double‐stranded DNA (Invitrogen) and visualized on a 1% agarose gel.

**TABLE 1:** Collection location of samples used in each pool, month and year of collection, latitude, longitude, and the number of individuals in collection (*n*)

Location	Month/year	Latitude	Longitude	*n*	Superpool	RD1	RD2
Adak Island	Mar. 2006	51.32	−176.23	48	AI	71	63
Kiska Island	Mar. 2005	51.89	177.47	48	AI	60	50
Kodiak Island	Mar. 2003	57.92	−152.30	46	EBS	70	62
Kodiak Island	Mar. 2005	57.92	−152.30	48	EBS	79	70
Near Island	Feb. 2005	52.60	174.40	47	AI	60	51
Pervenets Canyon (A)	Mar. 2016	59.21	−177.15	48	EBS	98	87
Pervenets Canyon (B)	Mar. 2016	59.21	−177.15	47	EBS	88	79
Pribilof Island	Apr. 2017	57.78	−172.12	48	EBS	84	76
Washington Coast (A)	Feb. 2005	48.27	−125.00	43	WA	87	81
Washington Coast (B)	Feb. 2005	48.27	−125.00	48	WA	81	66
Zhemchug Canyon	May 2017	58.27	−173.80	48	EBS	71	63

*Note*: Duplicate pools from Pervenets Canyon and Washington State were assigned to “A” and “B” collections because they were collected during the same year. Pools were grouped into three superpools, abbreviated as follows: Aleutian Islands (AI), Bering Sea (EBS), and Washington coast (WA). Mean read depth prior to filtering (RD1) and postfiltering (RD2) are presented, rounded to the nearest whole read.

DNA pools were constructed of 43–48 individuals with an equimolar concentration of DNA from each individual and a total of 750 ng of DNA per pool. In three spawning areas, we included DNA replicates to examine the accuracy of SNP calling. We included a temporal replicate sampled off Kodiak Island in 2003 (pool A) and 2005 (pool B) that were not genetically different at 11 microsatellites, with a statistically nonsignificant *F*_ST_ = −0.0006 (Cunningham et al., 2009). Two pools from Pervenets Canyon consisted of 95 individuals taken at a single location with longline gear were included to determine the effect of lower versus higher DNA concentration. These samples were split by DNA quantity into pool A (48 samples with the highest concentration of DNA, 47–72 ng/μl) and pool B (47 samples with the lowest DNA concentration, 25–47 ng/μl) to determine whether DNA concentration would affect Pool‐Seq results. Finally, two pools (A & B) of the Washington Coast samples contained the same 43 individuals, with five additional samples in pool B included to test sensitivity to 10% additional samples. These eight spawning locations with three replicates resulted in a total of eleven pools that were sequenced.

DNA libraries were prepared by and sequenced at the Northwest Genomics Center at the University of Washington (https://www.nwgs.gs.washington.edu/). Pooled samples were sheared using a Covaris LE220 focused ultrasonicator targeting an insert size of 350–380 base pairs (bp). The resulting sheared DNA was cleaned with Agencourt AMPure XP beads to remove sample impurities prior to library construction. A two‐sided AMPure cleanup was then performed in order to further restrict the fragment sizes to the desired range. End‐repair, A‐tailing, and ligation were performed as directed by the KAPA Hyper Prep Kit (KR0961 v1.14) protocol without amplification. A final AMPure cleanup was performed after ligation in order to remove excess adapter dimers from the library. All library construction steps were automated on a Janus Automated workstation (Perkin Elmer, Inc.).

Final library concentration prior to sequencing was determined by triplicate qPCR using the KAPA Library Quantification Kit (KK4824), and molecular weight distributions were verified using an Agilent Bioanalyzer (Agilent Technologies). Samples were sequenced on a HiSeq X using Illumina's HiSeq X Ten Reagent Kit (v2.5). Cluster generation was performed on a cBot modified for use with the HiSeq X flow cells, and flow cells were loaded onto the HiSeq X machine for sequencing.

### 2.2 Bioinformatics pipeline

The pipeline we used deviated from the standardized pipeline to account for pooled sequences, the nonmodel species, and the alignment of our sequence data to the reference genome of a related species (Atlantic cod). Within GATK (v4), we used the GenomeAnalysisToolkit v. 4.1.2.0 to identify a set of single‐nucleotide polymorphisms (SNPs) and to estimate allele frequencies for each pool. We designed and built a variant calling pipeline, including filtering for minimum and maximum read depth, missing data, and variant SNPs using the GATK best practices workflow (*germline short variant discovery*, *SNPs + Indels*, Poplin et al., 2018; Van der Auwera et al., 2013). Base calls generated on the HiSeq X instrument (RTA 2.7.6) were demultiplexed and converted to unmapped BAM files using the Picard programs ExtractIlluminaBarcodes and IlluminaBasecallsToSam in GATK. BAM files for each pool were then aligned to the Atlantic cod reference genome assembly, GadMor2 (Tørresen et al., 2017) using BWA‐MEM (Burrows‐Wheeler Aligner; v0.7.10). SNPs were identified using maximum likelihood expectation (MLE) to estimate the allele frequency rather than the observed read count (allele frequency) method in GATK v4. We assumed exactly 20 alleles per pool at each locus (ploidy), so that the minimum allele frequency for a variable SNP was 0.05 and the allele counts for each SNP summed to 20. This option was selected due to computational constraints and because maximum likelihood methodology was considered a good choice for allele frequency estimation. A single set of minimum and maximum read depth filters were applied to data that aligned to the GadMor2 linkage groups and scaffolds. A second set of filters was applied to remove SNPs exceeding set ranges for read depth per pool. The minimum read depth was 20× for the linkage group and scaffold data, and the maximum read depth was 2% of the total reads of each pool. The second filtering step removed any loci with missing data in any of the pools and retained only biallelic and variant SNPs. The full pipeline is described in more detail in the Appendix S1.

### 2.3 Assessing patterns of genetic differentiation

Lin's (1989, 2000) concordance correlation coefficient, *ρ*_*c*_, was calculated to measure relative agreement and validate results between the estimated allele frequencies among duplicated pools, using the R package *epiR* (Stevenson, 2020). More closely related pools were expected to have higher correlation. This statistic, which ranges from 0 to 1, with larger values indicative of higher concordance, compares allele frequency calls between two pools to determine deviation from perfect concordance:

$$\rho_{c}=\frac{2{\rho \sigma }_{X}\sigma_{Y}}{\sigma_{X}^{2}+\sigma_{Y}^{2}+{\left(\mu_{X}+\mu_{Y}\right)}^{2}},$$

where *μ*_x_ and *μ*_Y_ are the means of the allele frequencies in pools X and Y, and $\sigma_{X}$ and $\sigma_{Y}$ are the corresponding variances. The concordance correlation coefficient, *ρ*_*c*_, was calculated for the three duplicated pools (Washington, Pervenets, and Kodiak) to determine the relative correlation among them, as well as all other pools for comparison. The allele frequency matrix with the full filtered set of 1,944,780 variant SNPs was used for this analysis.

*F*_ST_ and *d*_XY_ were used to screen genomic regions of elevated divergence. *F*_ST_ is a relative measure of genetic differentiation (Wright, 1931) whose expectation is inversely correlated with genetic variation in one or both populations under comparison. Elevated pairwise *F*_ST_ may be due to lower within‐group genetic variation, rather than divergence among groups (Cruickshank & Hahn, 2014). Therefore, an absolute measure of divergence, *d*_XY_, whose expectation is independent of genetic variation was also used. The statistic *d*_XY_ summarizes the average number of nucleotide differences between pools of samples using an equation for unphased data: *d*_XY_ = (*p*_X_*q_Y_) + (*q*_X_*p_Y_), where *p*_X_ is the proportion of the reference allele in pool X and *p*_Y_ is the proportion of the reference allele in pool Y, *q*_X_ = 1 − *p*_X_, *q*_Y_ = 1 − *p*_Y_ (Dennenmoser et al., 2017; Schirrmann et al., 2018). Pairwise *F*_ST_ was calculated globally between all pools and per‐SNP between the three superpools described below (EBS, AI, and WA). *F*_ST_ was calculated in PoPoolation2 (Kofler et al., 2011) using Nei's (1973) formula,

$$F_{\mathrm{ST}}=\frac{{\hat{H}}_{T}-{\hat{H}}_{S}}{{\hat{H}}_{T}},$$

where ${\hat{H}}_{T}$ is the expected heterozygosity in combined pools/superpools and ${\hat{H}}_{S}$ is the expected heterozygosity in each pool/superpool (Hivert et al., 2018). The components ${\hat{H}}_{T}$ and ${\hat{H}}_{S}$ were calculated using Hivert et al. (2018), equations 17 and 18. While this measure of *F*_ST_ is considered biased (Hivert et al., 2018), the bias was consistent among pools because we retained similar read depth and sample size per pool (Table 1).

Pooled data were grouped into superpools according to the previously identified patterns of genetic differentiation between cod spawning in those regions (Drinan et al., 2018; Spies et al., 2020) and patterns identified in the principal components analysis (PCA) described below. Collections from Kodiak Island were included in the Bering Sea superpool based on these criteria. Three superpools were formed: the eastern Bering Sea superpool (“EBS”), the Aleutian Islands superpool (“AI”), and the Washington Coast (“WA”) superpool (Table 1). The Washington coast samples were designated as a third superpool due to their genetic distinctiveness. Allele frequencies for superpools were calculated as the arithmetic mean of the pool allele frequencies.

### 2.4 Sliding window analysis and islands of differentiation

A Gaussian kernel smoothing moving weighted average (KSMWA) approach was used to assess genome‐scale patterns of differentiation based on all identified loci. This analysis required pairwise comparisons between two sets of data; therefore, KSMWA was performed twice, on two pairs of superpools: (1) the Bering Sea superpool compared with the Washington coast superpool (EBS‐WA), and (2) the Bering Sea superpool compared with the Aleutian Islands superpool (EBS‐AI). This allowed for a relatively proximate, parapatric comparison (EBS‐AI) and a spatially distant, allopatric comparison (EBS‐WA) of genomic differentiation. Weighted average *F*_ST_ and *d*_XY_ was computed for all 29,825 consecutive windows along the genome for each superpool comparison.

We adapted the methodology of Hohenlohe et al. (2010) and Waters et al. (2018) for the KSMWA to accommodate pooled whole genome sequence data. The window was shifted along the genome by a step size that allowed the windows to overlap. We evaluated a range of step sizes from 7 to 50 kb and choices of window size sigma (*σ*) from 10 to 80 kb to balance smoothing the variation in *F*_ST_, the number of SNPs per window, and reducing noise along the window. The contribution of the SNP at position *p* to the region average was weighted by the Gaussian function *exp*(−[*p*‐*c*]^2^/2*σ* ^2^), where *c* is the window center and *σ* is a parameter for window size, so that SNPs in the center of the window received the highest weight according to a normal (*μ* = 0, *σ*) distribution. Sliding window analysis used a step size of 20 kb and *σ* = 30 kb, as described in the results section. The statistic *d*_XY_ was reported as the weighted average over all 180 k SNPs in each window, whereas weighted average *F*_ST_ was only calculated for variable positions, as it is undefined for invariable positions.

The significance of each sliding window's empirical Gaussian weighted average *F*_ST_ was determined by comparison to a set of bootstrap distributions generated from the entire genome (EBS‐AI or EBS‐WA). To reduce computational load, we generated a reference set of 2000 bootstrap distributions for each of the two KSMWA we performed. Each bootstrap distribution consisted of 10^6^ random draws with replacement of size *n* from the empirical per‐SNP *F*_ST_ estimates across the genome for that pairwise comparison, for *n* in 1–2000. One million bootstrap replicates were selected because this number sufficiently sampled the range of values of *F*_ST_. A Gaussian weighted average was calculated for each of the 10^6^ draws, arranged in random order. *p*‐values for each window were estimated as the proportion of bootstrap replicates higher or equal than the empirical weighted mean *F*_ST_'s. Statistics were adjusted for multiple tests using the Benjamini–Hochberg false‐discovery rate method (Benjamini & Hochberg, 1995).

Windows with kernel‐weighted *F*_ST_ averages considered significant based on the false‐discovery rate are hereafter referred to as *F*_ST_ outlier windows. Sliding window *d*_XY_ values were calculated in an analogous fashion.

Outlier regions with at least four consecutive windows with weighted mean *F*_ST_ >0.03 were considered islands of differentiation. The *F*_ST_ >0.03 threshold was selected because it contained the upper 10% of weighted average *F*_ST_ values, provided an inflection point above which *F*_ST_ values reached an asymptote, and was generally a threshold above which outlier windows appeared prominent (Figure S1). Outlier regions of interest were defined by the center points of full windows that shifted by step sizes of 20,000 bp. Therefore, the size of outlier regions was allowed to be smaller than full window sizes (180,000 bp). The coefficient of variation of the number of significant *F*_ST_ outlier windows per linkage group was also quantified for the EBS‐WA and the EBS‐AI superpool comparisons.

Weighted mean *d*_XY_ and *F*_ST_, and mean nucleotide diversity (*π*), and Tajima's D (*T*_*D*_, Tajima, 1989) were summarized over all *F*_ST_ outlier and nonoutlier windows for each KSMWA comparison (EBS‐AI and EBS‐WA) and for each EBS‐AI island of differentiation. Islands of differentiation for EBS‐WA comparisons were not included due to the much larger scale of differentiation among these superpools. For islands of differentiation between EBS‐AI superpools, we quantified the presence of windows with *d*_XY_ greater than the upper 5% quantile over all windows and windows with *d*_XY_ greater than the genome‐wide average. In early stages of population differentiation, *d*_XY_ may not be a reliable indicator of reduced gene flow (Cheng et al., 2021; Dennenmoser et al., 2017; Feulner et al., 2015). Therefore, for the EBS‐AI superpool comparison, Tajimas's D provided information on whether selection or reduced gene flow may be implicated in regions of increased divergence, and nucleotide diversity was used to assess genetic variation (Nei, 1986). Reduced nucleotide diversity (*π*) can result from strong directional selection, as well as background selection and selective sweeps (Booker & Keightley, 2018). Nucleotide diversity (*π*) was estimated in each of the 29,825 windows for each superpool as:

$$\pi =\sum \frac{2j\left(C-j\right)}{C\left(C-1\right)}/n,$$

where *j* is the derived allele count (*j* = the reference allele frequency × ploidy), *C* is the ploidy, or (20) × the number of pools in each superpool, summation takes place over all sites (including monomorphic sites) in each window, and *n* is the size of the window (180,000 bp). Tajima's D was also estimated in each sliding window as:

$$D=\frac{\pi -\theta_{W}}{\sqrt{\mathrm{var}\left(\pi -\theta_{W}\right)}},$$

where $\theta_{W}=\frac{S_{n}}{\sum_{i=1}^{C}\frac{1}{i}}$. Here, *S*_*n*_ = the number of SNPs in each sliding window. Note that each superpool had the same number of SNPs per window, but the number of SNPs per window varied.

Principal components analysis (PCA) was performed using all 1,944,780 SNPs in the full dataset using the R package *PCAdapt* (Luu et al., 2017) to visualize the genetic relationships among pools. The optimum number of principal components retained for analysis was determined based on Cattell's rule (Cattell, 1966).

We used *fst‐sliding*.*pl* in PoPoolation2 to estimate the global pairwise comparison of *F*_ST_ among all pools using the following options [*pool‐size 20*, *min‐count 1*, *min‐coverage 1*, *max‐coverage 500*, *window‐size 5,800,000*, *step‐size 0*]. For this calculation, the position for each SNP was renumbered sequentially and a window size that exceeded the total number of SNPs (*5,800,000)* was specified over this large region to calculate *F*_ST_ over the entire genome. The pool size was 20 alleles for each pool, reflecting a ploidy of 20. The option *min count 1* represents the minimum count of the minor allele and specifies that at least one pool must have an alternate allele to identify the position as a SNP, and SNPs were identified considering all populations simultaneously. Minimum and maximum coverage criteria applied to each pool were 1 and 500 reads, to ensure that no data were excluded. Secondly, PoPoolation2 *fst‐sliding*.*pl* was used to estimate the pairwise per‐SNP *F*_ST_ between superpools using the following options [‐‐*pool‐size 120:60:40 –min‐count 1 –min‐coverage 1 –max‐coverage 500 –window‐size 1 ‐‐step‐size 1*]. The pool size for that analysis reflected the number of pools per superpool multiplied by 20 (a ploidy of 120 for the EBS superpool, 60 for the Aleutian Islands superpool, and 40 for the Washington superpool).

### 2.5 Gene annotations

The 11 EBS‐AI islands of differentiation were aligned with all four GadMor2 annotation files (https://osf.io/4qsdw/; Tørresen et al., 2017) using the Bioconductor package *GenomicRanges* (Lawrence et al., 2013, Table 2). Only Bering Sea versus Aleutian Islands KSMWA comparisons were annotated, in line with the goals and hypotheses described above. Genes with known function were recorded if they contained one or more SNPs from the EBS‐AI dataset. The number of SNPs and mean *F*_ST_ over all SNPs within these proteins were also recorded, as well as a summary of gene function (www.uniprot.org).

**TABLE 2:** Number of outlier and nonoutlier windows, mean *F*_ST_, *d*_XY_, Tajima's D, and nucleotide diversity (*π*) for eastern Bering Sea (EBS) vs. Washington Coast (WA) comparisons (rows 1 and 2) and EBS vs. Aleutian Islands (AI, rows 3 and 4). Subsequent rows (3–15) provide these statistics for the 11 islands of differentiation between the EBS and AI superpools.

Row	Comparison	# win.	*F*_ST_	*d*_XY_	Tajima's D	*π*	CHL	SAL	TMP	VEL
	EBS vs. WA				D_EBS_/D_WA_	*π* × 10^−3^ (EBS/WA)	14,814	8848	7427	13,036
1	Nonoutlier	25,478	0.032	0.00054	−0.385/−1.179	0.547/0.530				
2	Outlier	4347	0.045	0.00059	−0.285/−1.230	0.573/0.525				
	EBS vs. AI	# win.	*F*_ST_	*d*_XY_ *(n sig*. *outliers)*	D_EBS_ (*n*)/D_AI_ (*n*)	*π* × 10^−3^ (EBS/AI)	CHL	SAL	TMP	VEL
3	Nonoutlier	28,996	0.017	0.00054	−0.370/−0.909	0.552/0.529				
4	Outlier	829	0.026	0.00053	−0.409/−1.000	0.529/0.492				
5	LG02_1	8	0.031	0.00058 (0)	1.446(0)/1.071(0)	0.499(0)/0.522(0)	1	12	1	8
6	LG06_1	10	0.025	0.00051 (0)	−0.556(0)/−1.438(4)	0.548(0)/0.450(0)	19	10	12	27
7	LG08_1	6	0.067	0.00029 (0)	−1.314(3)/−1.293(0)	0.184(5)/0.221(1)	1	29	3	38
8	LG08_2	3	0.023	0.00042 (0)	0.596(0)/ 0.260(0)	0.368(0)/0.384(0)	0	0	2	0
9	LG08_3	19	0.026	0.00029 (0)	−0.265(0)/−0.449(0)	0.252(2)/0.276(0)	1	10	3	16
10	LG12_1	47	0.049	0.00038 (0)	−0.522(3)/−0.730(0)	0.313(9)/0.339(7)	35	31	1	135
11	LG14_1	27	0.026	0.00027 (0)	−1.539(25)/−2.010(20)	0.272(2)/0.250(5)	8	11	5	18
12	LG16_1	12	0.030	0.00068 (2)	0.008(0)/−0.650(0)	0.663(0)/0.622(0)	0	3	2	1
13	LG18_1	13	0.025	0.00055 (0)	0.639(0)/−0.001(0)	0.527(0)/0.507(0)	1	1	3	10
14	LG19_1	5	0.044	0.00095 (0)	−0.310(0)/−1.225(0)	0.890(0)/0.741(0)	3	0	1	7
15	LG22_1	6	0.033	0.00069 (3)	0.370(0)/−0.085(0)	0.621(0)/0.626(0)	1	6	2	10

*Note*: Details on the 11 islands of differentiation also include the size of the island in number of windows (# win.), the number of windows in which *d*_XY_ was greater than the upper 5% quantile over all windows, in parentheses, mean Tajima's D (D_EBS_/D_AI_) and nucleotide diversity (*π*) from the EBS‐AI superpools followed by the number of windows in which Tajima's D or π was lower than the 5% quantile over all EBS‐AI windows. Gray‐shaded rows indicate outlier *F*_ST_ regions containing windows with *d*_XY_ greater than the genome‐wide mean *d*_XY_. Finally, the last four columns provide the number of SNPs for which log_10_ Bayes factor > 30 with regard to chlorophyll (CHL), salinity (SAL), temperature (TMP), and current velocity (VEL), presented over the entire genome for EBS‐WA and within islands of differentiation for EBS‐AI.

### 2.6 Environmental correlation

We selected four environmental covariates (salinity [psu], bottom temperature [°C], chlorophyll [mg/m^3^], and current velocity [m/s]) that are considered to be of impact to Pacific cod. Salinity, and particularly the interaction between salinity and temperature, affects growth, egg fertilization, and regulation of fish growth hormone during exposure to stress (Bœuf & Payan, 2001; Deane & Woo, 2009). Chlorophyll has been widely used as a proxy for marine productivity because it is indicative of phytoplankton biomass, and zooplankton, the prey of larval fish, forage on phytoplankton (Boyce et al., 2014; Hughes et al., 2018; Kristiansen et al., 2011). Recruitment in Pacific cod is more correlated with flow along and across the Bering Slope than other groundfish species, indicating that current velocity and direction are significant factors in Pacific cod early life history (Vestfals et al., 2014).

These four covariates were downloaded from the Copernicus Marine Environment Monitoring Services (CMEMS, https://resources.marine.copernicus.eu/), and values for each region were averaged over the cod spawning months January–April over the years in our study, 2003–2017. CMEMS is a global ocean eddy‐resolving (1/12° horizontal resolution, 50 vertical levels) reanalysis covering the period 1993 until present day. The reanalysis assimilates existing satellite and ocean vertical observations of temperature, salinity, and sea level and represents the state‐of‐the‐art in ocean models (Lellouche et al., 2018). Model simulations were extracted using the nearest grid point of the sample locations that was not on land with a “find nearest grid point” routine, which shifted the location by 4.5 km or less. The exception was the Adak Island sample for which the depth was outside the biological range of Pacific cod (3220 m); therefore, the sample location latitude was shifted slightly from 51.32° N to 51.62° N (33 km). Chlorophyll was extracted for surface values, but all other covariates were taken from the ocean bottom. Scalar current velocity was calculated from the east (*u*) and north (*v*) velocity components (m/s), $w=\sqrt{u^{2}+v^{2}}$.

We used BayPass v.2.2 (Gautier, 2015) to identify SNPs that were correlated to environmental variables within a Bayesian framework. The BayPass analysis was performed separately for the EBS‐WA and EBS‐AI comparisons, though for each comparison all pools were considered separately, not grouped as superpools. For the BayPass analyses, the Pribilof pool was excluded because it displayed a skewed allele frequency distribution with more SNPs with low minor allele frequencies than any other pool. The Pribilof sample was retained for all other analyses because the elevated number of low‐frequency SNPs had no noticeable effect on the allelic composition in superpools. The core BayPass model was run three times, with default parameters, to test for convergence. The auxiliary covariate model of BayPass associates allele frequencies with an environmental covariate while accounting for population structure. This was run with default settings, using the scaled mean values of the four covariates. To facilitate parallel computing, we used the function pooldata.subset() from the R package *poolfstat* (Hivert et al., 2018) to create 20 sets of 97,239 SNPs from our dataset. The auxiliary model was run three times for each covariate on the 20 sets of SNPs. The resulting per‐SNP X^T^X (a Bayesian measure of the deviation of population allele frequencies from expected values) and log_10_(Bayes factors) statistics were compared over replicated runs for each covariate by calculating Pearson's correlation coefficient among the three possible comparisons for each covariate, to ensure convergence before averaging values over runs. The average of the three scaled covariance matrices was used in the auxiliary covariate model. We considered all SNPs with an averaged log_10_(Bayes factor) in decibans (dB, the corresponding weights of evidence) greater than 30 associated with the covariate to be significant, a conservative threshold under which the alternative hypothesis is 10^30^ times more likely than the null hypothesis (Baldwin‐Brown & Long, 2020). We quantified the number of outlier SNPs in *F*_ST_ outlier windows for each covariate.

Finally, we plotted near‐bottom (2–7 m above the sea floor) optical depth for the Aleutian Islands and EBS shelf during summer (June–July) to evaluate differences in water clarity between regions during 2006, 2010, 2012, 2014, 2016, and 2018. Optical depth is a natural logarithmic ratio that characterizes how much downwelling irradiance just below the sea surface reaches a given depth. Optical depth was calculated from 6657 irradiance profiles obtained during NMFS bottom trawl surveys using light‐sensitive archival tags connected to the trawl net. Data were collected during years when both summer surveys (Aleutian Islands and EBS) were conducted and light data were collected. Methods for collecting irradiance data and calculating near‐bottom optical depth are described in Rohan et al. (2021). To quantify differences among regions, we fit a generalized additive model (GAM) to near‐bottom optical depth (response variable) using a penalized cubic regression spline of depth and region (Aleutian Islands or EBS shelf) as predictor variables. For a given depth, higher near‐bottom optical depth is associated with lower water clarity (i.e., less light transmission to the seafloor). Every 2.303 units of optical depth correspond with an order of magnitude difference in light transmission.

## 3 RESULTS

There were 13,993,143 SNPs in the raw dataset, which included 13,348,645 SNPs that aligned to the 23 GadMor2 linkage groups, 1740 SNPs that aligned to mitochondrial DNA, and 644,498 SNPs to scaffolds (Table A1). Initial average read depth per pool ranged from 60 to 98 (Table 1). Following filtering, the total number of SNPs that aligned to linkage groups was 1,944,780, while 161 SNPs aligned to mtDNA, and 21,546 SNPs aligned to scaffolds. Average read depth after filtering ranged from 50 to 87 per pool (Table 1). We retained the 1,944,780 SNPs that aligned to linkage groups for downstream analysis, hereafter referred to as the “full dataset”.

The concordance correlation coefficient showed that the highest similarity among all pairwise sample comparisons was between Washington Coast Pool A and Pool B (*ρ*_*c*_ = 0.9912383), followed by the Pervenets Pool A and Pool B (*ρ*_*c*_ = 0.9906341, Table A2). Samples taken in different years off Kodiak Island had the sixth highest concordance correlation coefficient (out of 46 comparisons), preceded by Pervenets Pool A compared with Kodiak Pool B, Zhemchug, and Kodiak Pool A, respectively (Table A2). Estimates of pairwise *F*_ST_ confirmed that Pool‐Seq genotypes were sensitive to the level of similarity among duplicate pools. Pairwise *F*_ST_ between duplicate pools increased in the inverse order as the *ρ*_*c*_, with lowest *F*_ST_ between Washington Coast pools A and B (*F*_ST_ = 0.0179), followed by Pervenets pools, (*F*_ST_ = 0.0181), and finally Kodiak (*F*_ST_ = 0.0205, Table 3). Estimates of *F*_ST_ between nearly duplicated pools are expected to be near zero, and elevated levels of pairwise *F*_ST_ among duplicated pools indicated some level of upward bias in *F*_ST_. The highest levels of pairwise *F*_ST_ were observed between the most distant comparisons, Near Island versus Washington Coast replicate pools A and B (*F*_ST_ = 0.0377, 0.0362), whereas more proximate samples exhibited smaller pairwise *F*_ST_ (e.g., Adak vs. Kiska *F*_ST_ = 0.0261). Therefore, *F*_ST_ appeared to be a robust measure of genetic differentiation in a relative sense, albeit positively biased.

**TABLE 3:** Pairwise *F*_ST_ between samples for all data combined, Wash. = Washington, Zhem. = Zhemchug, Perv. = Pervenets

	Near	Kiska	Adak	Perv. (A)	Perv. (B)	Zhem.	Pribilof	Kodiak 2003	Kodiak 2005	Wash. (A)
Near	–									
Kiska	0.0250	–								
Adak	0.0262	0.0261	–							
Perv. (A)	0.0229	0.0228	0.0238	–						
Perv. (B)	0.0236	0.0235	0.0245	0.0181	–					
Zhemchug	0.0249	0.0248	0.0257	0.0197	0.0204	–				
Pribilof	0.0250	0.0248	0.0258	0.0195	0.0202	0.0219	–			
Kodiak 2003	0.0248	0.0245	0.0255	0.0199	0.0204	0.0216	0.0219	–		
Kodiak 2005	0.0243	0.0242	0.0250	0.0193	0.0200	0.0212	0.0215	0.0205	–	
Wash. (A)	0.0377	0.0371	0.0364	0.0317	0.0322	0.0334	0.0333	0.0321	0.0314	–
Wash. (B)	0.0362	0.0358	0.0361	0.0298	0.0303	0.0315	0.0316	0.0301	0.0296	0.0179

**FIGURE 2:**
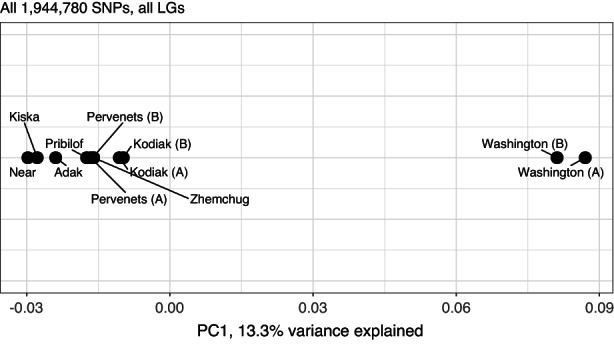
Principal components analysis for all pools and all (1,944,870) SNP loci. One principal component was the optimal choice to represent the data, which explained 13.3% of the variance; plots are shown along a single axis only.

A PCA generated with all 1,944,780 filtered loci was optimized using only one principal component, which explained 13.3% of the variance, as indicated by Cattell's rule applied to the scree plot (Figure S2). The Aleutian Islands and Bering Sea samples clustered together, with Aleutian Islands pools to the left of Bering Sea pools (Figure 2). Aleutian Islands samples ordered by longitude in the PCA, with the furthest west, Near Islands, to the left, followed by Kiska, then Adak. The Bering Sea pools were closely grouped, and following the longitudinal pattern, Kodiak samples were furthest to the right of the cluster that included Pervenets, Pribilof, and Zhemchug. Washington pools were distant, and some separation was observed between the two Washington pools.

### 3.1 Sliding window analysis and islands of differentiation

For the *F*_*ST*_ sliding window analysis, a step size of 20 kb and *σ* = 30 kb was selected for the Gaussian kernel smoothing moving weighted average (KSMWA) because it optimized between SNPs per window, variation in *F*_ST_, and reduced noise within windows (Figure S3 and Table A3). This resulted in 29,825 windows.

**FIGURE 3:**
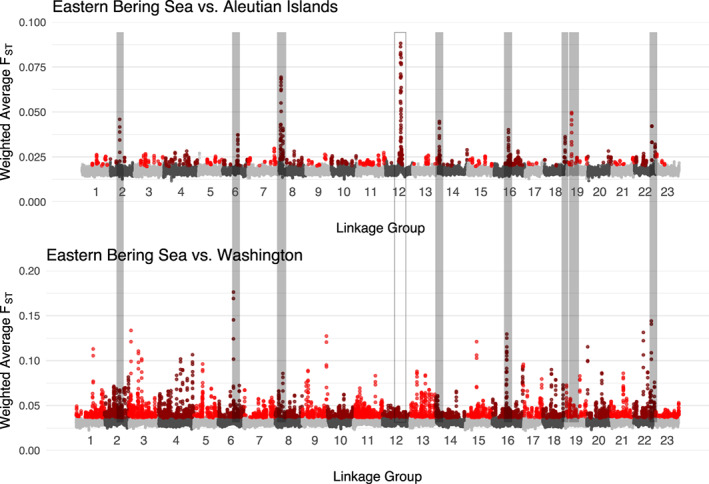
Weighted average *F*_ST_ for all SNPs aligned to GadMor2 in 180 kbp windows that overlap with a step size of 20,000 bp between the Bering Sea and Aleutian Islands superpools (upper panel), and the Bering Sea and Washington Coast superpools (lower panel). *F*_ST_ outlier windows are colored in red shades. Islands of differentiation identified in Table 2 are highlighted with gray bars. The island on linkage group 12 is not filled because it was not present in the EBS‐WA comparison.

**FIGURE 4:**
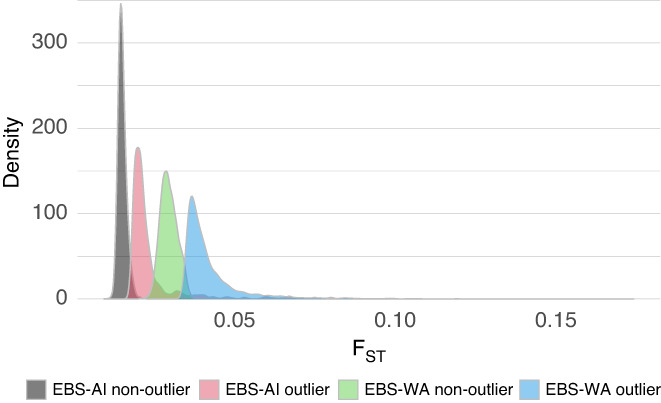
Density plot of normalized *F*_ST_ within and outside *F*_ST_ outlier windows for the Bering Sea vs. Aleutian Islands comparison (EBS‐AI) and the Bering Sea vs. Washington comparison (EBS‐WA).

The pattern and extent of *F*_ST_ outlier windows differed between the EBS‐AI and the EBS‐WA superpool comparisons; the EBS‐AI comparison showed fewer outlier windows (829) than the EBS‐WA comparison (4347), and the highest EBS‐WA outlier *F*_ST_ windows peaked at *F*_ST_ = 0.2, approximately double that of the highest EBS‐AI outlier windows (Figure 3). Furthermore, the coefficient of variation of the number of outlier windows per linkage group was higher for the EBS‐AI comparison (CV = 0.026) than for EBS‐WA (CV = 0.015), indicating more uneven distribution of outlier regions in the EBS‐AI comparison. The distribution of weighted average *F*_ST_ values for all windows was higher in EBS‐WA than in EBS‐AI outlier and nonoutlier windows, indicative of a generally higher *F*_ST_ between allopatric than parapatric Pacific cod (Table 2, Figure 4). In contrast, mean *d*_XY_ was identical between EBS‐WA and EBS‐AI comparisons in nonoutlier regions, and slightly higher (0.00059 vs. 0.00053) in the EBS‐WA comparisons than in the EBS‐AI comparison (Table 2).

Mean Tajima's D was generally negative across all outlier and nonoutlier regions across all EBS‐AI and EBS‐WA superpool comparisons, but lower in outlier regions versus nonoutlier regions (Table 2). Tajima's D was similar between the EBS among outlier versus nonoutlier regions (EBS‐WA comparison), but remained negative. Nucleotide diversity, π, was lower in the EBS and Aleutian Islands outlier than nonoutlier regions, and EBS pools showed higher nucleotide diversity than Washington coast or Aleutian Island pools, particularly on LG06 and LG19 islands of differentiation (Table 2).

The largest EBS‐AI *F*_ST_ outliers were on linkage groups (LG) 8 and 12, and smaller islands of differentiation were present on linkage groups 2, 6, 14, 16, 18, 19 and 22 (Table 2, Table A4, Figure 3). In one of these 11 islands of differentiation (LG16), *d*_XY_ exceeded the upper 5% quantile of observed over all EBS‐AI in 2/10 windows, and in five islands of differentiation (LG02_1, LG12_1, LG16_1, LG18_1, and LG22_1), *d*_XY_ exceeded the genome‐wide average (Table 2). Most islands of differentiation with elevated *d*_XY_ showed no evidence for significantly reduced Tajima's D, with the exception of LG12_01, the largest *F*_ST_ outlier window, in which three windows (position 15,300,000–15,520,000) contained reduced Tajima's D. This region of reduced Tajima's D occurred at one end of the window, whereas the region of high *d*_XY_ occurred at the other end. Reduced Tajima's D was observed at LG06_1 in the Aleutian Islands (in 4/10 windows) and at LG08_1 in the EBS samples (in 3/6 windows). Reduced nuclear diversity was present in both the EBS (5/6) and AI (1/6) at LG08_1, although the results do not rule out background selection in both regions. In LG14_1, there was reduced Tajima's D in 25 out of 27 EBS windows and 20 out of 27 AI windows.

### 3.2 Gene annotations

**FIGURE 5:**
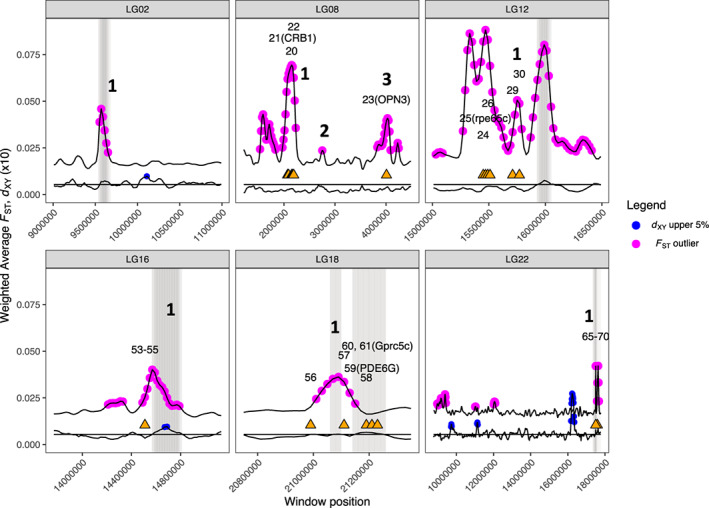
Weighted average *F*_ST_ and *d*_XY_ (the latter multiplied by 10 for visualization) in the six linkage groups containing *F*_ST_ outlier regions: linkage groups 2, 8, 12, 16, 18, and 22, labeled corresponding to *F*_ST_ outlier regions in Table 2. Shaded vertical lines represent outlier regions within which linkage groups with *F*_ST_ outliers are greater than 0.030 and pairwise *d*_XY_ is greater than the genome average (0.000539 × 10, horizontal black line). Blue points represent the upper 5% quantiles for *d*_XY_, and pink dots indicate *F*_ST_ outlier windows. Orange triangles represent locations of annotated genes within *F*_ST_ outlier windows, which are labeled corresponding to the gene number in Table A5, and vision gene names are listed.

Within the 11 islands of differentiation, we identified 68 regions with similarity to known genes (Table 4 and Table A5, Figure 5). The genes were responsible for a variety of functions, but notably there were five genes related to vision were identified within *F*_ST_ outlier regions (Table A5): CRB1 (LG8_1), OPN3 (LG08_3), rpe65c (LG12_1), PDE6G (LG18_1), and Gprc5c (LG18_1). PDE6G and Gprc5c were located adjacent to the *F*_ST_ outlier on LG18 in a region of above‐average *d*_XY_ and were therefore considered potentially relevant.

**TABLE 4:** Annotated genes within which *F*_ST_ outlier SNPs regions were found

LG, number	Region (Mb)	Gene name	Description
8_01	2.18–2.19	CRB1	Plays a role in photoreceptor morphogenesis in the retina
8_03	4.03–4.04	OPN3	G‐protein coupled receptor which selectively activates G proteins via ultraviolet A (UVA) light‐mediated activation in the skin. Opsins are light‐absorbing genes
12_1	15.47–15.48	rpe65c	Retinal Mueller cells isomerohydrolase. Catalyzes forms of retinoic acid to meet the high demand for the chromophore by cones
18_1	21.22–21.23	PDE6G	Retinal rod rhodopsin‐sensitive cGMP participates in transmission, amplification of visual signal
18_1	21.24–21.25	Gprc5c	Retinoic acid‐inducible G‐protein coupled receptor; RA shapes the developing eye and is essential for normal optic vesicle and anterior segment formation

*Note*: This table represents a subset of the 68 gene regions; a complete list is in Table A5.

### 3.3 Environmental correlation

Salinity, temperature, current velocity, and chlorophyll patterns differed among regions (Figure S4 and Table A6). The lowest chlorophyll was typically found in Bering Sea sampling areas, Zhemchug and Pervenets. Salinity was highest at the Washington Coast site and lowest off Kodiak. Temperature was highest off the Washington Coast (~8°C) and lowest in Bering Sea sites, 2–3°C. The highest current velocities were found in the Aleutian Islands, with Adak the highest, followed by Near and Kiska. BayPass results were significantly correlated over each set of three independent runs (Table A7); therefore, results were averaged over each set of three runs. Among the EBS‐AI comparisons, the most notable increase in Bayes factors in regions identified as islands of differentiation was found in linkage group 12 ([Link], [Link]). Tabulation of the correlates most strongly correlated with allele frequencies indicated that in LG12_1, velocity had the largest effect, with 135 SNPs with Bayes factors >30 (Table 2). Velocity was the biggest covariate in LG08_1, with 38 SNPs strongly correlated. The second most significant correlate after velocity was salinity, with 29 strongly correlated SNPs in LG08_1 (Table 2). In the EBS‐WA BayPass analysis, results were tabulated over all data due to the increased number of high *F*_ST_ regions. In this comparison, the highest number of strongly correlated Bayes factor SNPs was correlated with salinity (36%) and velocity (32%), versus temperature (20%) and chlorophyll (12%, Table 2).

**FIGURE 6:**
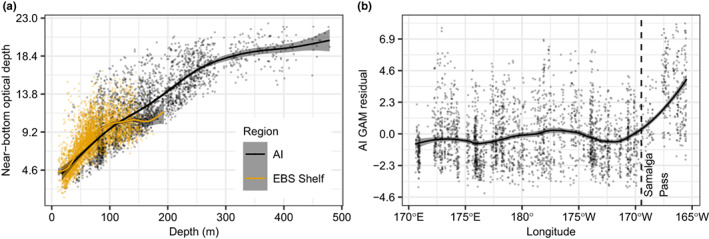
Generalized additive model (GAM) fitted between depth (meters) and near‐bottom optical depth data in the Aleutian Islands (AI) and eastern Bering Sea from summer (June–July) 2006–2018. Panels show: (a) GAM fitted mean (line) ± 1 standard error (shading), (b) AI GAM residuals by longitude, mean residual based on LOESS regression (line) ± 1 standard error (shading), and position of Samalga Pass (~169°29′W). Point and line colors denote region (AI and EBS).

The EBS shelf has lower water clarity than the Aleutian Islands at ~20–160 m bottom depths during summer, based on near‐bottom optical depth, meaning that less surface‐incident irradiance reaches the seafloor in the EBS than in the Aleutian Islands at those depths (Figure 6a). Based on GAM fitted means, the maximum difference in optical depth between the EBS and Aleutian Islands is at ~70 m depth, where light transmission to the seafloor in the Aleutian Islands is slightly more than an order of magnitude higher than the EBS (difference in near‐bottom optical depth: 2.45). In addition, there is an east–west break in GAM residuals in the Aleutian Islands around Samalga Pass (169°28′W), where bottom optical depth is higher (“darker”) than predicted to the east of Samalga Pass than to the west (Figure 6b).

## 4 DISCUSSION

While Pacific cod from the Aleutian Islands and EBS are sufficiently genetically differentiated to merit separate management units (Drinan et al., 2018; Spies, 2012; Spies & Punt, 2015), there is little information on the mechanisms driving these differences. We examined how genomic patterns of differentiation and environmental covariates differed among these proximate and distant comparisons, inferred processes by which differentiation occurs across the genome, and examined genetic factors that may play a role in divergence within islands of differentiation between Aleutian Island and Bering Sea cod. A primary finding was a pattern of heterogeneous genomic differentiation between Pacific cod from the Aleutian Islands and EBS. We identified 11 genomic islands of differentiation in nine (of 23) linkage groups between the proximate EBS and Aleutian Islands, and the presence of many more *F*_ST_ outlier regions between the EBS and Washington coast (Table 2; Figure 3). The EBS‐AI islands of differentiation ranged in size from 60,000 to 940,000 bp, and weighted average *F*_ST_ over these 11 islands ranged from 0.023 to 0.067 (Figure 3; Table A4). The limited number of EBS‐AI islands of differentiation interspersed with regions of low *F*_ST_ were consistent with emergent local adaptation among populations experiencing migration–selection balance, similar to our first hypothesis, although alternative explanations and limitations are discussed below.

High *F*_ST_ and elevated *d*_XY_ in five of the 11 genomic islands, particularly on LG12 and LG16 (Figure 5; Table 2), may indicate local adaptation or inversions (Lotterhos, 2019), but the Pool‐Seq data preclude further examination of the nature of the outlier regions. There was evidence for selection in the Aleutian Islands region of LG06_1 and in the EBS region of LG08_1. In LG08_1, reduced nucleotide diversity in the EBS and Aleutian Islands could alternatively be a result of background selection or directional selection with hitchhiking in both regions. In LG14_1, reduced Tajima's D and reduced nucleotide diversity provided evidence for background selection, although it is not always possible to distinguish between background selection and directional selection with hitchhiking (Booker & Keightley, 2018). Higher nucleotide diversity in EBS pools could be a result of selection on Aleutian Islands or Washington Coast cod, or due to the disproportionately larger size of the EBS cod stock, as larger population sizes may lead to higher nucleotide diversity (Spies et al., 2020; Subramanian, 2019).

The pairwise *F*_ST_ between the EBS and Washington coast was roughly double that between the Aleutian Islands and Bering Sea spawning populations (Table 3; Figure 3). This may be consistent with a framework in which gene flow acts to homogenize allele frequencies between EBS and Aleutian Islands at most of the genome, except the few regions affected by selection, while lower gene flow or longer time since divergence between the EBS and Washington Coast allows for *F*_ST_ to increase. However, the similar absolute divergence (*d*_XY_) in nonoutlier regions and slightly smaller *d*_XY_ in the EBS versus Aleutian Islands outlier regions compared with EBS versus Washington indicate a similar number of nucleotide differences and are consistent with a scenario in which EBS and Aleutian Islands populations began diverging soon after they colonized the region following the recession of the last glacial maximum (Canino et al., 2010).

Most EBS‐AI *F*_ST_ outlier regions were also present in the EBS vs. Washington comparison (Figure 3), indicating that these EBS‐AI outlier regions may be important for EBS cod. Regions of elevated differentiation due to hitchhiking with genes under selection start as smaller regions that grow into wider regions over time and tend to be more pronounced in small populations where linkage is higher (Feder & Nosil, 2010). In contrast, islands of differentiation that are the result of genomic inversions are more likely to emerge in high gene flow species with strong selective pressure (Schaal et al., 2022). The divergent region on linkage group 12, the most predominant island of differentiation with the highest EBS‐AI mean *F*_ST_, was not present in the EBS‐WA comparison, suggesting that this outlier region may have developed relatively recently if EBS and AI populations diverged more recently (Figure 3).

While there is no clear evidence for the mechanism leading to the island of differentiation observed on LG12 in Pacific cod, it is slightly less than 1 megabases in size, much smaller than four large inversions that have been identified in Atlantic cod between Northeast Arctic and Norwegian Coastal ecotypes (Berg et al., 2017; Sodeland et al., 2016). In Atlantic cod inversions on linkage groups 1, 2, 7, and 12 were 17, 5, 9.5, and 13 megabases long, respectively (Kirubakaran et al., 2016; Sodeland et al., 2016). While the island of differentiation in Pacific cod is on the same linkage group as one inversion in Atlantic cod (linkage group 12), it appears located between 15 and 16 mB and is outside the range suggested by previous studies (0.4–0.6 mB—14mB) which aligned to GadMor2 (Barth et al., 2017), and GadMorCeltic (Kirubakaran et al., 2016).

We hypothesized that temperature would be the most significant environmental correlate to genomic variation; however, current velocity and vision stood out as potential factors leading to islands of differentiation but temperature did not. Strong correlation with velocity based on BayPass results indicated that genes located within linkage group 12 outliers were associated with different environments of the EBS and AI, although the precise relationship is not clear (Table 2, Figure S4). Furthermore, the finding of five genes related to vision in outlier regions differentiating the Aleutian Islands and Bering Sea may be an indication of the genes underlying selection. However, identifying targets of selection within islands of differentiation is difficult because the lack of recombination, whether due to inversions or selection with hitchhiking, can link true targets selection with false positives (Berg et al., 2017). Visual systems have been shown to be under strong natural selection in several fish species including Atlantic cod in which differential expression of visual opsins and the rhodopsin *rhI* gene varies by ecotype (Berg et al., 2017; Hofmann & Carleton, 2009; Pampoulie et al., 2015; Valen et al., 2018). In other species, visual systems have evolved in response to environment; for example, the visual system of Midas cichlids (*Amphilophus* cf. *citrinellus*) has rapidly evolved to adapt to the clear water in crater lakes from more ancestral turbid lakes (Torres‐Dowdall et al., 2017).

Our analysis of near‐bottom optical depth suggests there are depth‐associated differences in water clarity among cod habitat in the EBS and Aleutian Islands, as well as differences within the Aleutian Islands chain itself (Figure 6). The break in water clarity conditions at Samalga Pass is presumably due to higher concentrations of chlorophyll (Mordy et al., 2005), chromophoric dissolved organic matter, and nonalgal particulate in the Alaska Coastal Current than in the more ocean‐influenced waters to the west. Specific genes identified in outlier regions for Pacific cod included CRB1, which plays a role in photoreceptor morphogenesis in the retina, two genes related to retinoic acid (rpe65c and Gprc5), a gene related to retinal rod rhodopsin (PDE6G) and an opsin receptor (OPN3), and are consistent with life history strategies and adaptation associated with vision (Table 4). While the association between vision genes and islands of differentiation in Pacific cod is not evidence for causation, research in other teleosts and the commonality with Atlantic cod in which vision genes are differentially expressed among ecotypes provides a basis for further exploration of potential effects of foraging, predator avoidance, orientation, and social behavior (Hofmann & Carleton, 2009; Valen et al., 2018).

The data passed multiple checks, confirming the reliability Pool‐Seq methodology for genotyping. Biological replicate pools have been used in other studies too as a quality check for accurate sample contribution and allele frequency calling (Dorant et al., 2019; Guirao‐Rico & González, 2021; Hivert et al., 2018). Read depth (50–100) was within the range that is considered sufficient for resolution of the allele frequency spectrum, distinguished evolutionary patterns, and provided sufficient power for Tajima's D (Ferretti et al., 2013), while the number of samples per pool (43–48) was sufficient to reduce experimental bias (Dorant et al., 2019; Kofler et al., 2011). High relative agreement in allele frequencies among the Washington Coast pools was consistent with expectations that pools consisting of the same or similar individuals would produce highly correlated allele frequencies. The PCA also confirmed similarity among duplicate and proximate samples (Figure 2), although allele frequencies differed between the two Washington replicated pools by only adding five individuals (Figure 2). In the case of the Pervenets pools, high correlation indicated that pooling based on concentration of DNA within the observed ranges did not impact the results. *F*_ST_ estimates decreased with increasing concordance correlation coefficients, providing a second line of evidence for genotype similarities among pools (Table 3). The concordance correlation coefficient between the two Kodiak samples taken in different years was relatively high (6th highest out of 55 comparisons), but was lower than several comparisons between Kodiak and EBS spawning cod. The similarity between Kodiak and Pervenets samples is confirmed by pairwise *F*_ST_; *F*_ST_ between temporal replicates from Kodiak were higher (indicating less similar) than any pairwise comparisons between Kodiak 2003/2005 and Pervenets Pool A or B. We do not completely understand the level of connectivity between western Gulf of Alaska spawning cod and those from the Bering Sea shelf, although the PCA is consistent with isolation by distance observed in previous studies (Figure 2; Cunningham et al., 2009). Similarly, several previous studies found similarity among Kodiak samples and Unimak spawning cod from the EBS (Table A2; Cunningham et al., 2009; Drinan et al., 2018).

While this was the first population genomics study of Pacific cod using whole genome sequencing, the pooled WGS platform posed some drawbacks. Without individual‐level data, there was no insight into linkage disequilibrium, which could provide evidence for genomic inversions. Further, ploidy was limited to 20 per pool, so differences among pools could only be observed in increments of 0.05, and subtle differences among pools may have been lost, although this is unlikely given the large number of SNPs. High replicability between pools provided confidence that the underlying measures of population allele frequencies were accurate. While the patterns observed in the PCA appear sound, the nature of Pool‐Seq limited the number of principal components in the data to 10, and the resulting PCA was optimized with only a single principal component that explained 13.3% of the variance. We also acknowledge that our analyses were performed over large window sizes (*σ* = 30 kb, step size = 20 kb), which could reduce the possibility of finding the effects of single genes. Another concern was that while alignment to the genome of a congeneric species is routine among nonmodel species, chromosomal rearrangements may not have aligned and could have been missed. Therefore, we anticipate that our approach laid the groundwork for future studies to examine outlier regions in Pacific cod in more detail. Future work that includes low coverage whole genome sequencing (lcWGS) on an individual basis will provide further understanding of the outlier regions identified here, as well as identifying whether outlier regions represent inversions or regions of linked selection (Lou et al., 2021).

Overall, data provided new evidence for heterogeneous differentiation across the genome between spawning populations of cod from the EBS and Aleutian Islands, which we hypothesize may be the result of local adaptation despite some low level of gene flow. Furthermore, we found more extensive levels of relative differentiation but similar levels of absolute divergence among cod from the allopatric EBS vs. Washington coast. These results indicate that the EBS and Aleutian Islands may have started diverging soon after cod recolonized the North Pacific following the last glacial maximum. The presence of 11 islands of differentiation, five of which showed some level of elevated *d*_XY_, and evidence for directional selection and background selection on two other islands of differentiation indicate that local adaptation has occurred between cod from these different environments. Results also indicate that these spawning groups are in migration–selection balance and that selection may be strong enough to balance the effects of gene flow. While our study was not designed to provide direct evidence of genes responsible for adaptation, annotated genes suggested that vision may play a role in adaptation to the distinct ocean environments of the Bering Sea and Aleutian Islands. Results build upon previous studies indicative of divergence between EBS and Aleutian Islands Pacific cod.

## CONFLICT OF INTEREST

The authors declare that they have no conflicts of interest.

## Supporting information


Figure S1.

Figure S2.

Figure S3.

Figure S4.

Figure S5a.

Figure S5b.

Table S5.

Table S6.

Appendix S1.

## Data Availability

The data that support the findings of this study are openly available in the Sequence Read Archive (SRA), which is accessible from National Center for Biotechnology Information (NCBI) at https://www.ncbi.nlm.nih.gov/sra/, reference number PRJNA675289 (University of Washington, 2021).
