# Supplementary figures and images for: Genomic differentiation in Pacific cod using Pool‐Seq
Source: Evol Appl. 2022 Oct 13;15(11):1907–24. doi: 10.1111/eva.13488 (PMC9679252; doi:10.1111/eva.13488)

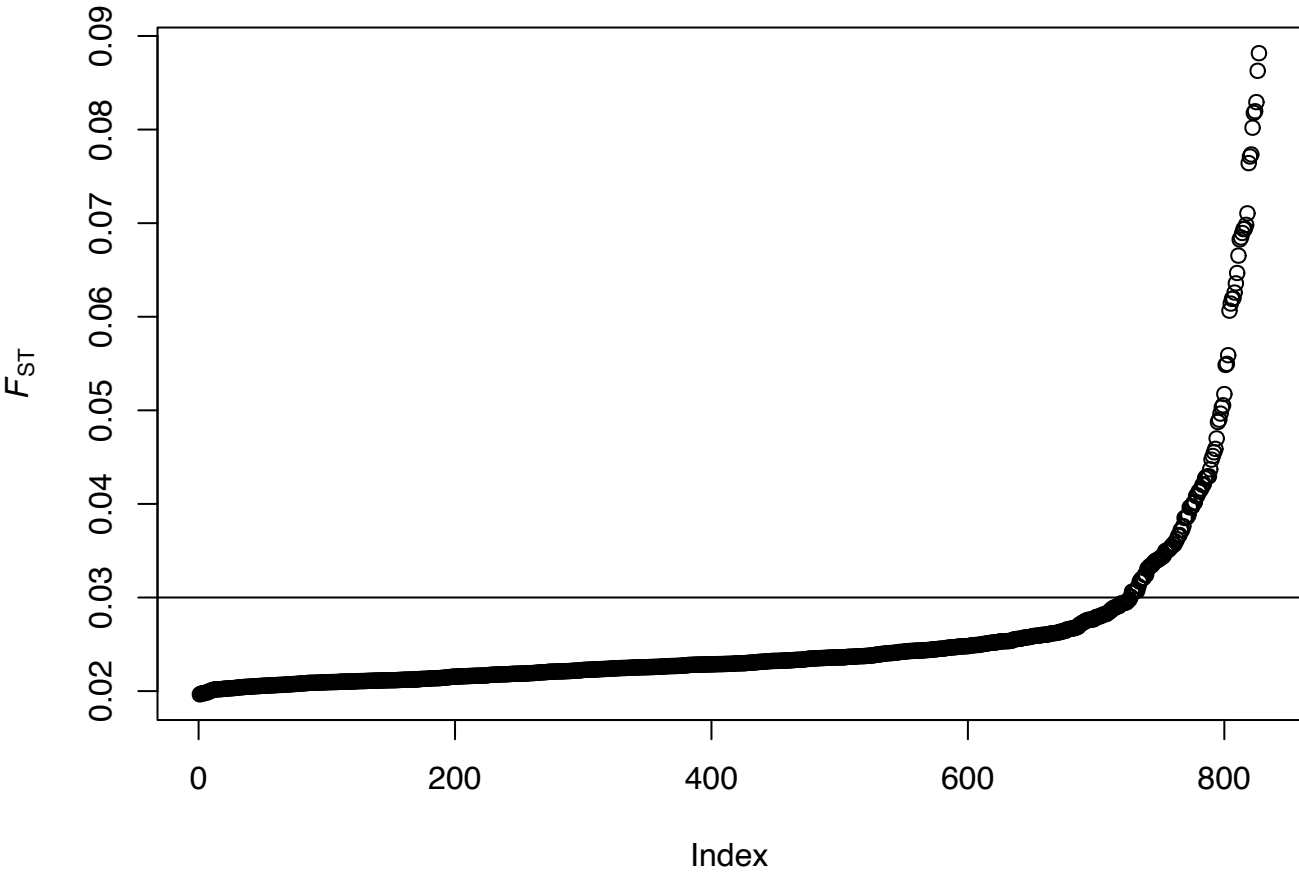

Supplement: Supplementary file 1 — Figure S1. [file EVA-15-1907-s001.pdf]

Scree Plot - K = 10

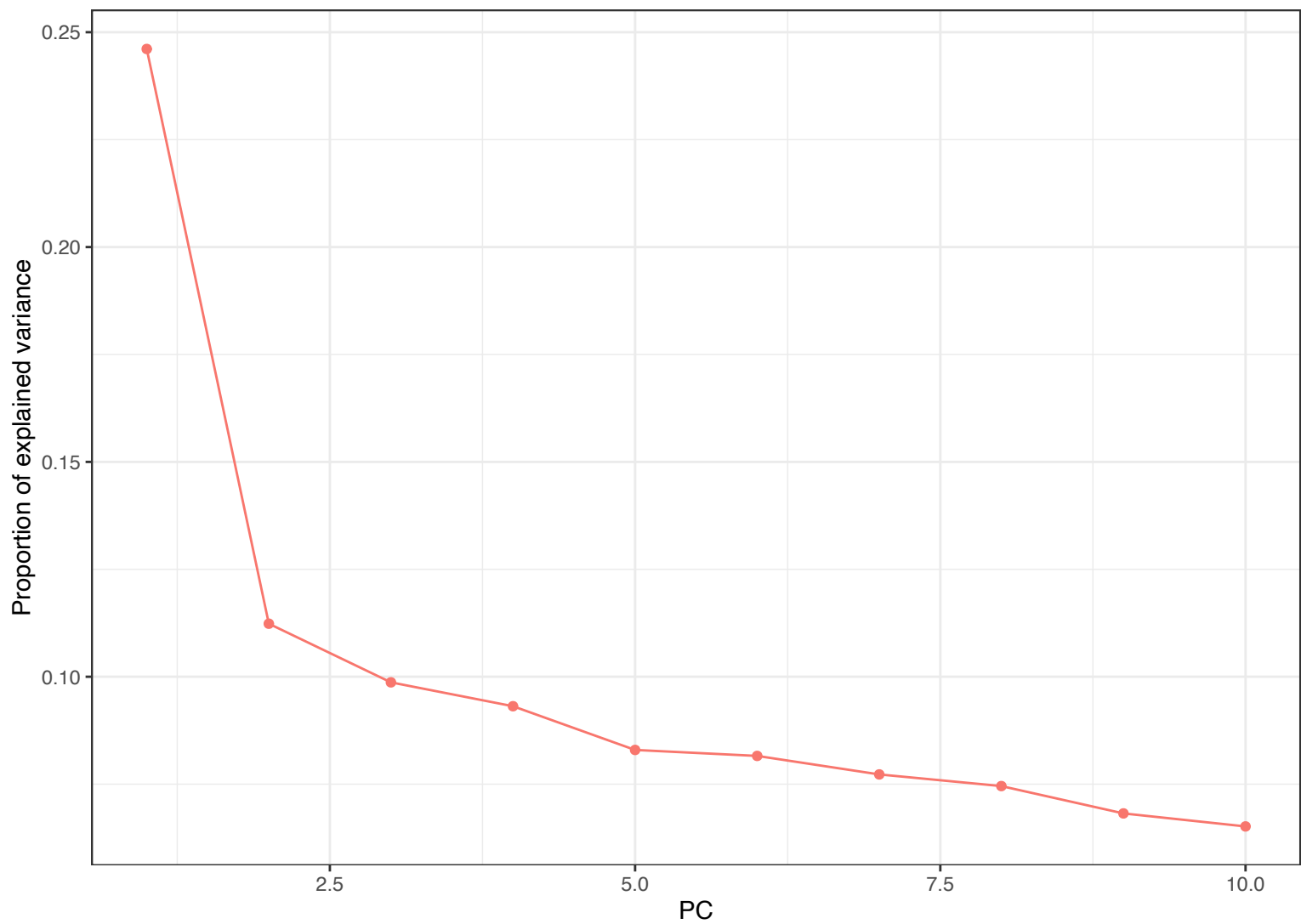

Supplement: Supplementary file 2 — Figure S2. [file EVA-15-1907-s008.pdf]

Bayes Factors >30, ALU & EBS pools, blue bars show KSMWA FST ALU/EBS outlier windows

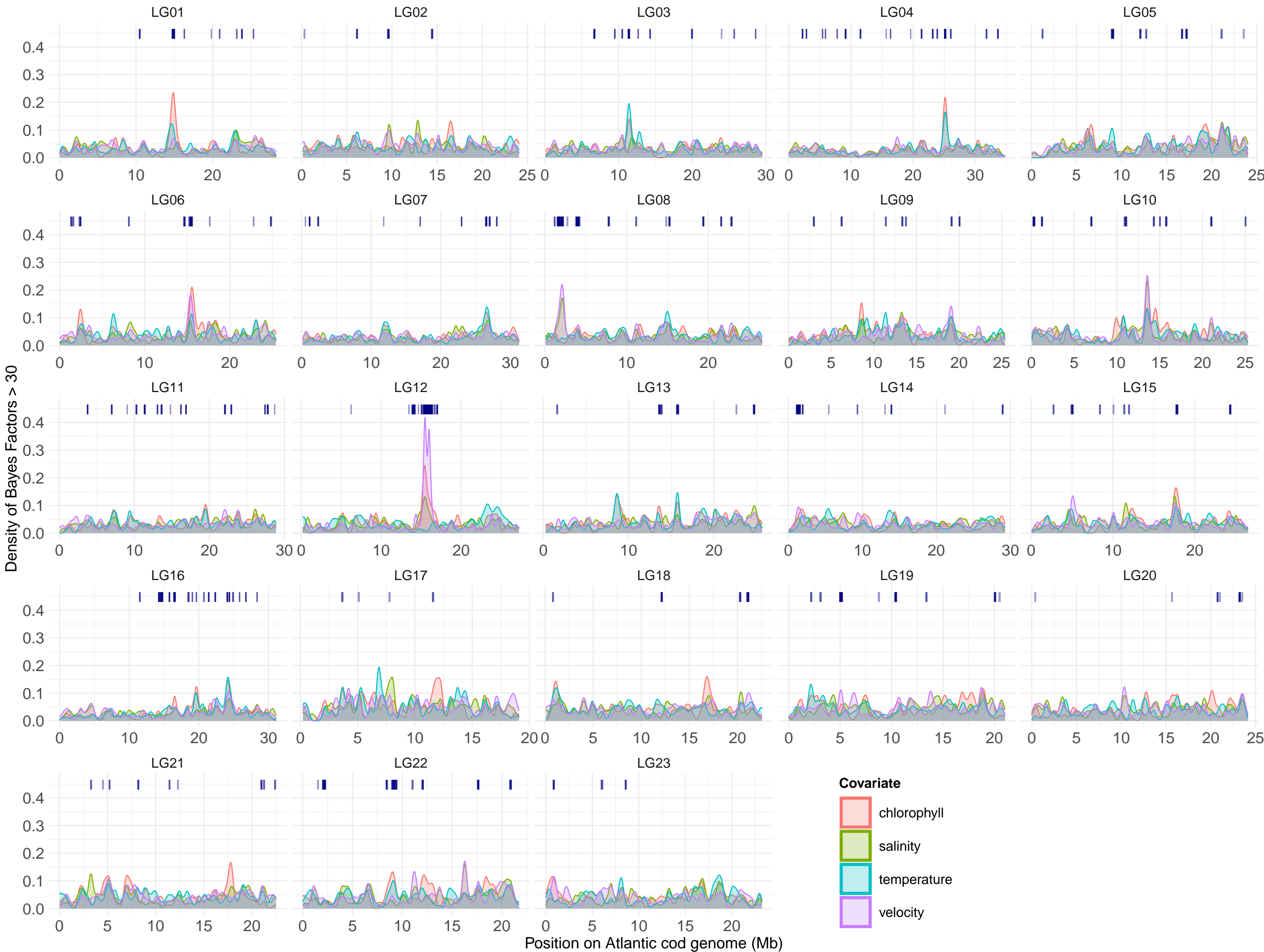

Supplement: Supplementary file 5 — Figure S5a. [file EVA-15-1907-s005.pdf]

Bayes Factors >30, EBS & WA pools, blue bars show KSMWA WA/EBS FST outlier windows

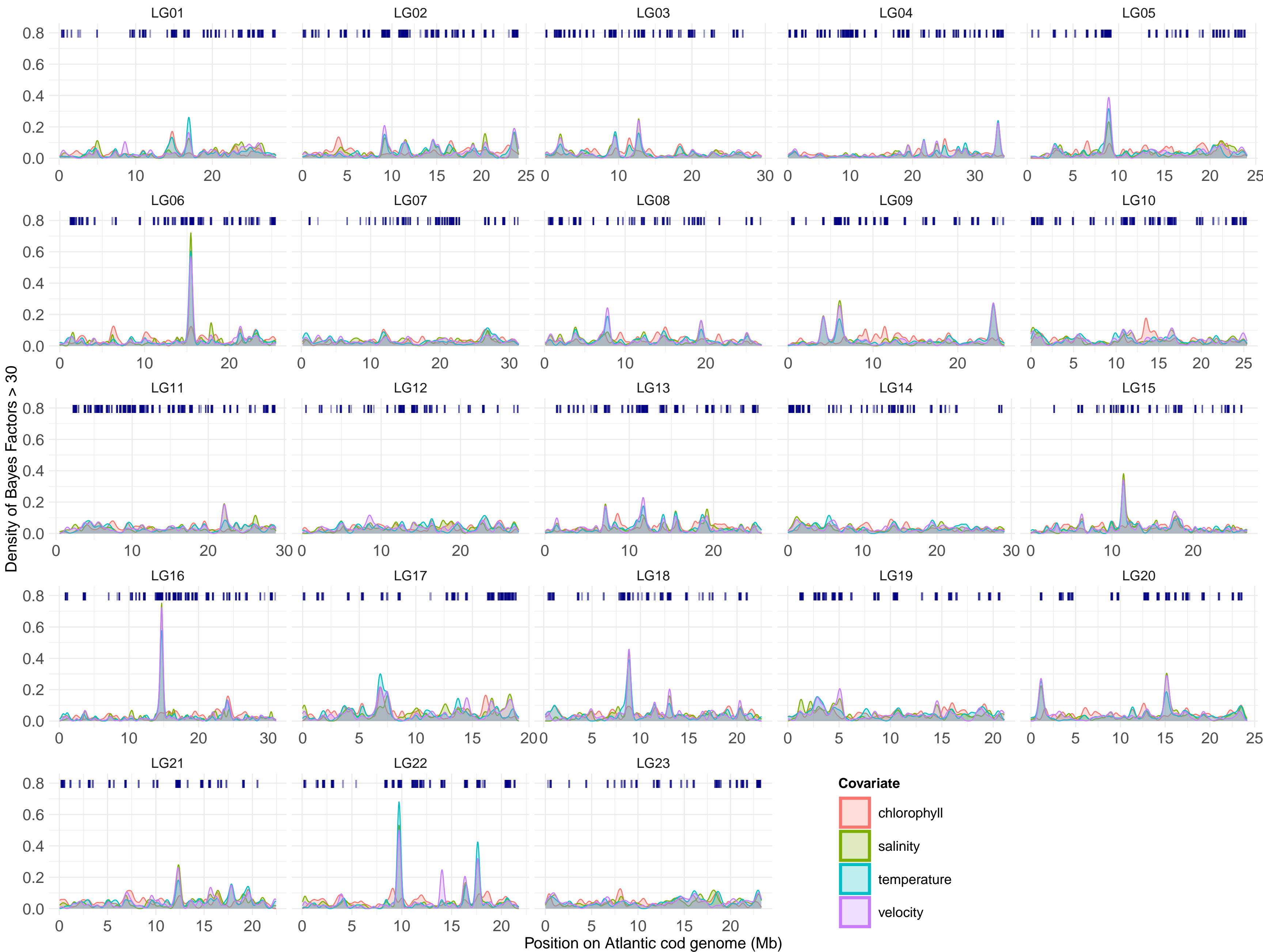

Supplement: Supplementary file 6 — Figure S5b. [file EVA-15-1907-s009.pdf]
