# Supplementary material for: Genomic differentiation in Pacific cod using Pool‐Seq
Source: Evol Appl. 2022 Oct 13;15(11):1907–24. doi: 10.1111/eva.13488 (PMC9679252; doi:10.1111/eva.13488)

Figure A3 (A): Eastern Bering Sea vs. Aleutian Islands kernel smoothing moving weighted average F_{ST}, LG01 (black line, left axis title) for a range of sigma (σ, 10-80 kb) and step sizes (7-50 kb). The number of SNPs per window is represented by a red line, right axis title. Note that axis titles (left, right, and x-axis) apply to all panels.

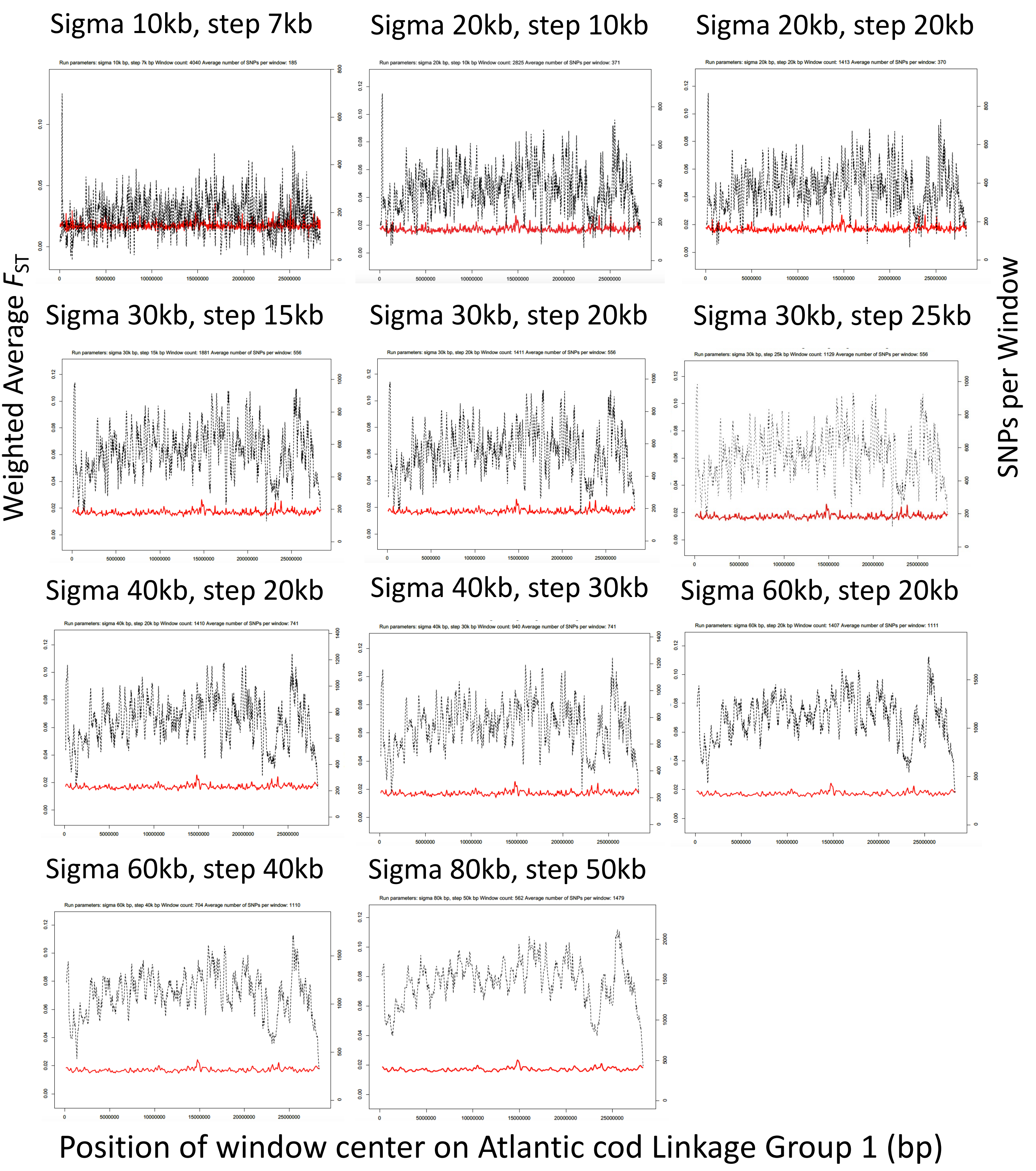

Figure A3 (B): Eastern Bering Sea vs. Washington kernel smoothing moving weighted average F_{ST}, LG01 (black line, left axis title) for a range of sigma (σ, 10-80 kb) and step sizes (7-50 kb). The number of SNPs per window is represented by a red line, right axis title. Note that axis titles (left, right, and x-axis) apply to all panels.

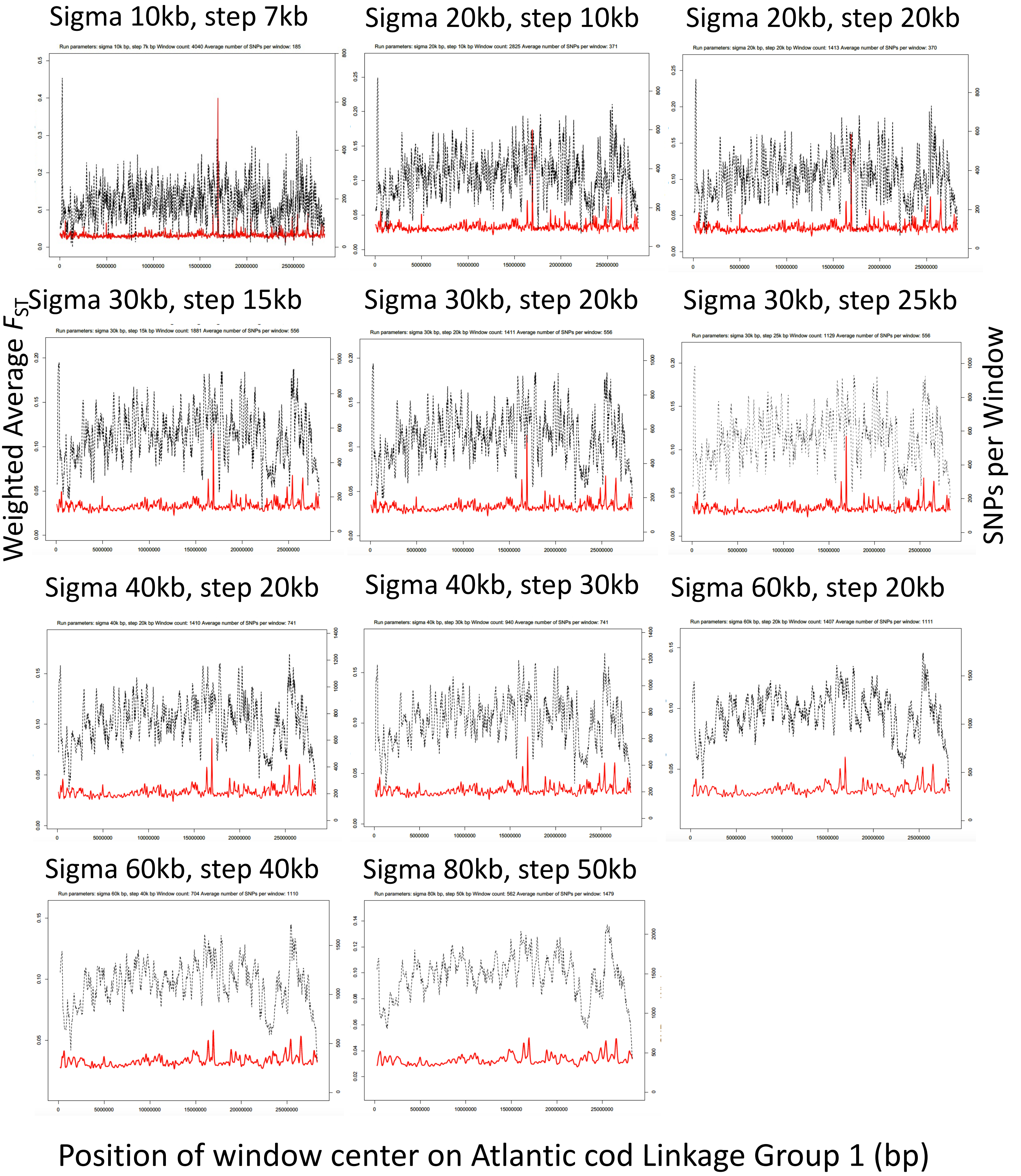

Supplement: Supplementary file 3 — Figure S3. [file EVA-15-1907-s006.pdf]
