# Supplementary material for: Genomic differentiation in Pacific cod using Pool‐Seq
Source: Evol Appl. 2022 Oct 13;15(11):1907–24. doi: 10.1111/eva.13488 (PMC9679252; doi:10.1111/eva.13488)
Supplement: Supplementary file 4 — Figure S4. [file EVA-15-1907-s007.pdf]

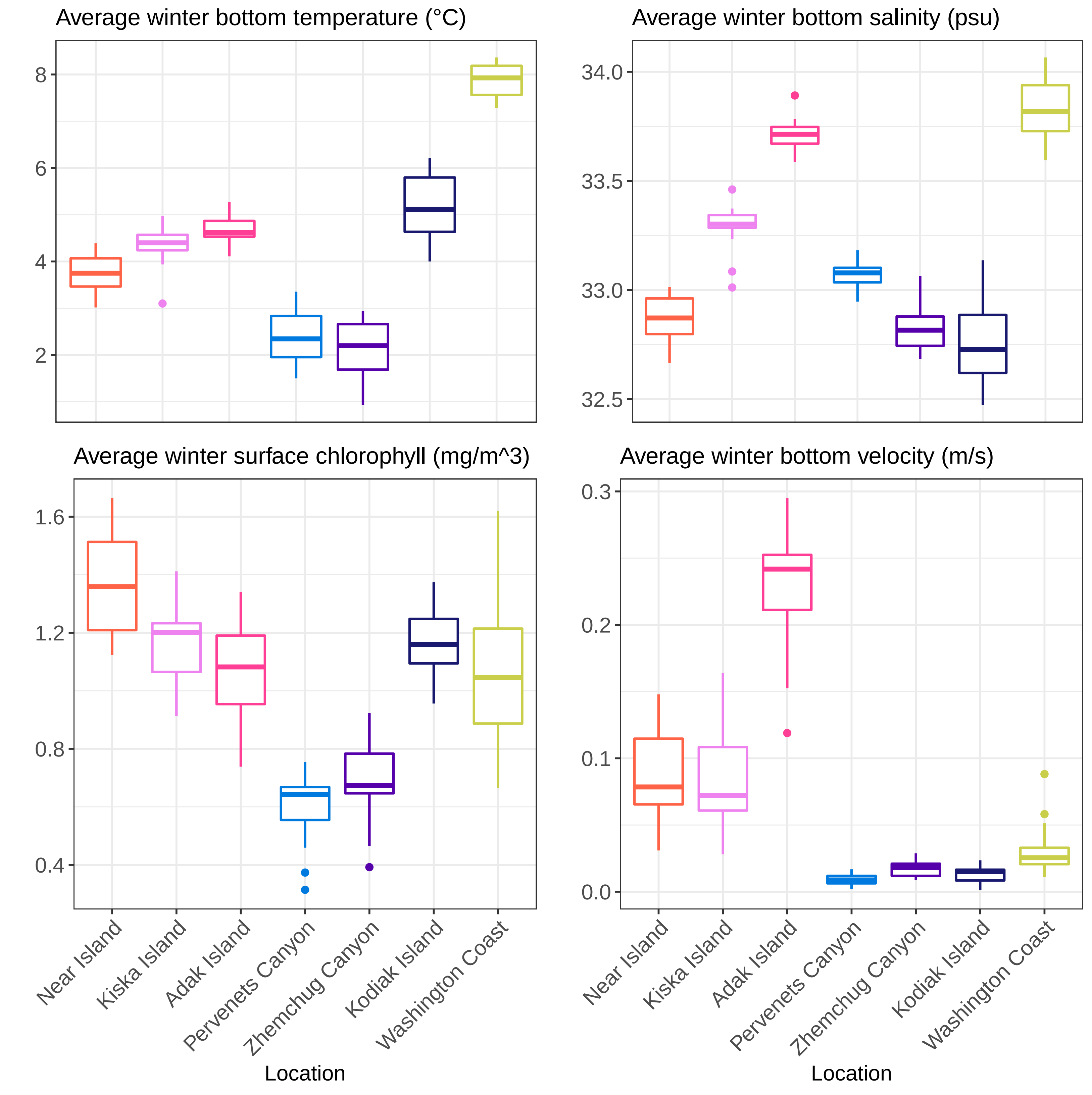

Average winter bottom temperature (°C)
8
6
4
2
Average winter bottom salinity (psu)
34.0
33.5
33.0
32.5
Average winter surface chlorophyll (mg/m^3)
1.6
1.2
0.8
0.4
Average winter bottom velocity (m/s)
0.3
0.2
0.1
0.0
Near Island
Kiska Island
Adak Island
Pervenets Canyon
Zhemchug Canyon
Kodiak Island
Washington Coast
Location
