# Supplementary material for: Genomic differentiation in Pacific cod using Pool‐Seq
Source: Evol Appl. 2022 Oct 13;15(11):1907–24. doi: 10.1111/eva.13488 (PMC9679252; doi:10.1111/eva.13488)
Supplement: Supplementary file 9 — Appendix S1. [file EVA-15-1907-s002.docx]

Appendix: Customized Variant Calling with GATK for pooled whole genome sequence data

The GenomeAnalysisToolkit (GATK v4), pipeline was used to identify variant SNPs and filter for minimum and maximum read depth and missing data (McKenna et al. 2010). We adapted the standardized pipeline for Pool-Seq data (Van der Auwera et al. 2013). The GATK program HaplotypeCaller (v4.1.2.0) was used in genomic variant call format (GVCF) mode to identify and flag potentially variant sites in each of the pools (McKenna et al. 2010, DePristo et al. 2011), with the following argumets: [--pcr-indel-model NONE, --sample-ploidy 20, --max-alternate-alleles 3, --min-pruning 3, --max-genotype-count 1771, --read-filter OverclippedReadFilter]. The options [--disable-read-filter NotDuplicateReadFilter and --pcr-indel-model NONE] were used because any duplicate reads (reads with the same start location) could be from different individuals, as PCR was not used in the library preparation. Although the true ploidy of our samples was between 92 and 96, a sample ploidy of 20 was used because the highest ploidy that GATK could computationally handle (in v4.1.2.0) was 21. To reduce the computational load, we only allowed the identification of two alternate alleles beyond the reference allele [--max-alternate-alleles 3, --min-pruning 3]. The argument [--max-genotype-count 1771] refers to the maximum number of genotypes mathematically possible at a locus. The number of possible genotypes is N_G_=(*g*+*m*-1)!/(*g*-1)!*m*!, where *g* = ploidy and *m* is the number of possible alleles (Haldane 1948). With a ploidy of 20 and 4 possible alleles (three selected plus one for missing data), N_G_= (20+4-1)!/(4-1)!(20)! = 1,771. Finally, we used the OverclippedReadFilter program to remove reads that were potentially from foreign organisms. For each pool, we left-aligned and trimmed the common bases from the indels using LeftAlignAndTrimVariants so they were minimally represented, using arguments: [--split-multi-allelics FALSE, --dont-trim-alleles FALSE, --max-indel-length 500].

We used the GATK process Joint Genotyping, where all pools are evaluated together to call variants, because it allowed for more sensitive variant detection and higher accuracy. The linkage groups and mitochondrial data for each pool were imported into their own Genomics Data Base using GenomicsDBImport. The linkage groups were too large to analyze in their entirety for all pools at the same time, so we used the scatter/gather option to run the pipeline iteratively over the 23 linkage groups and mitochondrial data. The linkage groups and mitochondrial data were split into intervals of 10^6^ bp, and each interval was genotyped for all pools concurrently using GenotypeGVCFs. Options used to run GenotypeGVCFs over linkage groups matched those used with HaplotypeCaller [--max-alternate-alleles 3, --sample-ploidy 20, --max-genotype-count 1771] as well as [--only-output-calls-starting-in-intervals TRUE]. Iteratively analyzed sections of the genome were subsequently combined into vcf files with the GatherVcfsCloud program and SNPs were retained with the program SelectVariants.

While sequences located on the linkage groups (LGs) were genotyped jointly, the scaffolds were treated separately. There were too many scaffolds (8,262) to use the Genomics Data Base method to combine the data from each pool. Instead, using scatter-gather, the scaffolds from each pool were merged into one GVCF per scaffold with CombineGVCFs and the combined gVCF files were then genotyped with GenotypeGVCFs. The resulting VCF files were combined with GatherVCFs into one VCF file that was sorted with SortVCF, indexed with IndexFeatureFile and SelectVariants was used to select only SNPs.

SNPs were removed that did not meet quality thresholds using GATK. Those quality thresholds were ["QD < 2.0" (quality normalized by depth of non reference samples),"SOR > 3.0" (strand bias), "FS > 60.0", Fisher's test to detect strand bias (Fisher 1922), "MQ < 55.0" (root of the mean square of mapping quality of the reads across all samples), "MQRankSum < -12.5 (Mann-Whitney rank sum test for the mapping quality), and "ReadPosRankSum < -8.0" (distance from the end of the read for the alternate allele)]. The single set of minimum and maximum read depth filters applied to data that aligned to the GadMor2 linkage groups and scaffolds is described in the main text. A different set of filter limits were used for the mitochondrial data (Table S7). The minimum and maximum read depth for mitochondrial data were twice the standard deviation of the mean read depth per pool.

References for Appendix

DePristo, M.A., Banks, E., Poplin, R., Garimella, K.V., Maguire, J.R., Hartl, C., Philippakis, A.A., Del Angel, G., Rivas, M.A., Hanna, M. and McKenna, A., 2011. A framework for variation discovery and genotyping using next-generation DNA sequencing data. *Nature genetics*, *43*(5), p.491.

Haldane, J.B.S., 1948. The number of genotypes which can be formed with a given number of genes. *Journal of genetics*, *49*(2), pp.117-119.

McKenna, A., Hanna, M., Banks, E., Sivachenko, A., Cibulskis, K., Kernytsky, A., Garimella, K., Altshuler, D., Gabriel, S., Daly, M. and DePristo, M.A., 2010. The Genome Analysis Toolkit: a MapReduce framework for analyzing next-generation DNA sequencing data. *Genome research*, *20*(9), pp.1297-1303.

Van der Auwera, G.A., Carneiro, M.O., Hartl, C., Poplin, R., Del Angel, G., Levy‐Moonshine, A., Jordan, T., Shakir, K., Roazen, D., Thibault, J. and Banks, E., 2013. From FastQ data to high‐confidence variant calls: the genome analysis toolkit best practices pipeline. *Current protocols in bioinformatics*, *43*(1), pp.11-10.

Figure Captions for Appendix

Figure S1. Distribution of weighted average *F*_ST_ in EBS-AI outlier windows. Horizontal line represents *F*_ST_ = 0.03.

Figure S2. Scree plot showing in decreasing order the percentage of variance explained by each principal component, for all 1,944,780 SNPs in the full dataset.

Figure S3. Weighted average *F*_ST_ for linkage group 1 (EBS-AI) shown for step sizes from 7 kb to 50 kb and sigma (σ) from 10 kb to 80 kb.

Figure S4. Values for chlorophyll (mg/m^3^), salinity (psu), temperature (°C), and velocity (m/s) averaged over the spawning months January – April during years 2003-2017 for the stations shown, at locations based on Table 2 as described in the methods.

Figure S5. Manhattan plot of BayPass SNPs with Bayes Factors (>30) (very strong evidence) in the Bering Sea vs. Aleutian Islands comparison (panel A), and Bering Sea vs. Washington Coast (panel B). Regions that were identified as high *F*_ST_ outlier windows in the EBS-AI (panel A.) and EBS-WA comparisons (panel B.) are shaded as vertical bars. The environmental correlates (current velocity, salinity, chlorophyll, and temperature) associated with each SNP are provided in the legend.

Appendix Tables

Table S1. Summary of the number of variant bases identified with GATK Joint Genotyping, the number of SNPs retained after quality filtered, and after all filters were applied.

| Genomic position | Variant bases | Quality filtered SNPs | Fully filtered SNPs |
| --- | --- | --- | --- |
| LG01 | 591,847 | 264,755 | 87,320 |
| LG02 | 560,859 | 238,715 | 79,309 |
| LG03 | 660,549 | 281,156 | 96,347 |
| LG04 | 849,101 | 327,898 | 117,542 |
| LG05 | 507,161 | 220,539 | 71,653 |
| LG06 | 559,956 | 252,022 | 85,653 |
| LG07 | 732,955 | 307,544 | 105,124 |
| LG08 | 635,354 | 260,666 | 87,486 |
| LG09 | 563,076 | 264,342 | 85,487 |
| LG10 | 580,138 | 260,637 | 89,885 |
| LG11 | 621,245 | 279,843 | 93,937 |
| LG12 | 597,004 | 260,133 | 83,497 |
| LG13 | 564,962 | 260,540 | 83,673 |
| LG14 | 640,630 | 303,390 | 102,481 |
| LG15 | 608,540 | 252,728 | 86,779 |
| LG16 | 703,533 | 290,850 | 100,474 |
| LG17 | 423,010 | 160,384 | 54,945 |
| LG18 | 487,939 | 223,954 | 71,847 |
| LG19 | 464,235 | 214,938 | 71,059 |
| LG20 | 534,297 | 234,448 | 80,824 |
| LG21 | 483,449 | 211,095 | 67,823 |
| LG22 | 467,834 | 205,888 | 69,544 |
| LG23 | 509,231 | 214,560 | 72,091 |
| LG total | 13,346,905 | 5,791,025 | 1,944,780 |
| MT_genome | 1,740 | 427 | 161 |
| Scaffolds | 644,498 | 41,712 | 21,546 |
| Combined Total | 13,993,143 | 5,833,164 | 1,966,487 |

Table S2. Concordance correlation coefficient (*ρ_c_*) of allele frequencies for the 1,944,780 variant SNPs in the full dataset, in descending order, with 95% confidence intervals (CI). The *ρ_c_* was calculated for all pairwise sets of pools. Duplicated pools for Washington, Pervenets, and Kodiak are referred to as “A” and “B”, as defined in Table 1 and comparison among duplicated pools are highlighted.

| Pool1 | Pool2 | *ρ_c_* | Lower CI | Upper CI |
| --- | --- | --- | --- | --- |
| Washington A | Washington B | 0.9912383 | 0.9912241 | 0.9912525 |
| Pervenets A | Pervenets B | 0.9906341 | 0.9906189 | 0.9906493 |
| Pervenets A | Kodiak B | 0.99017 | 0.9901541 | 0.9901859 |
| Pervenets A | Zhemchug | 0.9899673 | 0.9899511 | 0.9899836 |
| Pervenets A | Kodiak A | 0.9899506 | 0.9899343 | 0.9899669 |
| Kodiak A | Kodiak B | 0.9899028 | 0.9898864 | 0.9899191 |
| Pervenets B | Kodiak B | 0.989895 | 0.9898786 | 0.9899114 |
| Kodiak B | Washington B | 0.989895 | 0.9898786 | 0.9899114 |
| Pervenets B | Kodiak A | 0.9897227 | 0.989706 | 0.9897393 |
| Pervenets B | Zhemchug | 0.989628 | 0.9896112 | 0.9896448 |
| Pervenets A | Pribilof | 0.9895734 | 0.9895565 | 0.9895903 |
| Zhemchug | Kodiak B | 0.9894958 | 0.9894787 | 0.9895128 |
| Zhemchug | Kodiak A | 0.9893408 | 0.9893235 | 0.989358 |
| Pervenets B | Pribilof | 0.9892552 | 0.9892378 | 0.9892726 |
| Pribilof | Kodiak B | 0.98881 | 0.9887919 | 0.9888281 |
| Pribilof | Washington B | 0.98881 | 0.9887919 | 0.9888281 |
| Pribilof | Kodiak A | 0.9886203 | 0.9886019 | 0.9886387 |
| Kiska | Pervenets A | 0.9885772 | 0.9885587 | 0.9885957 |
| Zhemchug | Pribilof | 0.9885466 | 0.9885281 | 0.9885651 |
| Zhemchug | Washington B | 0.9885466 | 0.9885281 | 0.9885651 |
| Near | Pervenets A | 0.9885081 | 0.9884895 | 0.9885266 |
| Pervenets A | Washington B | 0.9885081 | 0.9884895 | 0.9885266 |
| Kiska | Pervenets B | 0.9882816 | 0.9882626 | 0.9883005 |
| Kiska | Washington B | 0.9882816 | 0.9882626 | 0.9883005 |
| Near | Pervenets B | 0.9882146 | 0.9881955 | 0.9882336 |
| Pervenets B | Washington B | 0.9882146 | 0.9881955 | 0.9882336 |
| Kiska | Kodiak B | 0.9881969 | 0.9881777 | 0.9882159 |
| Near | Kodiak B | 0.9881188 | 0.9880996 | 0.988138 |
| Kiska | Kodiak A | 0.9880837 | 0.9880644 | 0.988103 |
| Near | Kiska | 0.9880239 | 0.9880045 | 0.9880433 |
| Near | Kodiak A | 0.9879649 | 0.9879454 | 0.9879844 |
| Adak | Pervenets A | 0.9879309 | 0.9879113 | 0.9879504 |
| Kiska | Zhemchug | 0.9878822 | 0.9878626 | 0.9879018 |
| Near | Zhemchug | 0.9877854 | 0.9877657 | 0.9878052 |
| Adak | Kodiak B | 0.9876566 | 0.9876367 | 0.9876766 |
| Adak | Pervenets B | 0.987652 | 0.987632 | 0.9876719 |
| Adak | Kodiak A | 0.9874744 | 0.9874541 | 0.9874947 |
| Kiska | Adak | 0.987368 | 0.9873476 | 0.9873885 |
| Near | Adak | 0.9873153 | 0.9872948 | 0.9873358 |
| Adak | Zhemchug | 0.9872654 | 0.9872447 | 0.987286 |
| Kiska | Pribilof | 0.9872448 | 0.9872241 | 0.9872654 |
| Near | Pribilof | 0.9871563 | 0.9871355 | 0.987177 |
| Adak | Pribilof | 0.9866162 | 0.9865946 | 0.9866379 |
| Kodiak A | Washington B | 0.9851439 | 0.9851198 | 0.9851679 |
| Pribilof | Washington A | 0.984341 | 0.9843157 | 0.9843663 |
| Kodiak B | Washington A | 0.984341 | 0.9843157 | 0.9843663 |
| Kodiak A | Washington A | 0.9840664 | 0.9840406 | 0.9840921 |
| Pervenets A | Washington A | 0.9837044 | 0.983678 | 0.9837307 |
| Pervenets B | Washington A | 0.9835326 | 0.983506 | 0.9835592 |
| Zhemchug | Washington A | 0.9832547 | 0.9832276 | 0.9832817 |
| Near | Washington B | 0.9822699 | 0.9822413 | 0.9822985 |
| Adak | Washington B | 0.9821557 | 0.9821269 | 0.9821845 |
| Adak | Washington A | 0.9819037 | 0.9818745 | 0.9819329 |
| Kiska | Washington A | 0.981695 | 0.9816655 | 0.9817245 |
| Near | Washington A | 0.9813668 | 0.9813367 | 0.9813968 |

Table S3. A range of sigma and step sizes were analyzed to balance smoothing the variation in *F*_ST_, the number of SNPs per window, and reducing noise along the window (see also Appendix Figure 1). The number of windows (window count) and the (average) number of SNPs per window for a range of step sizes from 7 kb to 50 kb and choices of sigma (*σ*) from 10 kb to 80 kb are shown. Note: results are the same for each pair of superpools. A step size of 20 kb and *σ* = 30 kb were selected.

| Sigma | step size | Window count | SNPs per window |
| --- | --- | --- | --- |
| 10,000 | 7,000 | 4,040 | 185 |
| 20,000 | 10,000 | 2,825 | 371 |
| 20,000 | 20,000 | 1,413 | 370 |
| 30,000 | 15,000 | 1,881 | 556 |
| 30,000 | 20,000 | 1,411 | 556 |
| 30,000 | 25,000 | 1,129 | 556 |
| 40,000 | 20,000 | 1,410 | 741 |
| 40,000 | 30,000 | 940 | 741 |
| 60,000 | 20,000 | 1,407 | 1,111 |
| 60,000 | 40,000 | 704 | 1,110 |
| 80,000 | 50,000 | 562 | 1,479 |

Table S4. Genomic ranges of *F*_ST_ outlier windows.

| *F*_ST_ outlier | Window start | Window stop | Length (bp) |
| --- | --- | --- | --- |
| LG02_1 | 9,500,000 | 9,660,000 | 160,000 |
| LG06_1 | 15,300,000 | 15,700,000 | 400,000 |
| LG08_1 | 2,080,000 | 2,200,000 | 120,000 |
| LG08_2 | 2,720,000 | 2,780,000 | 60,000 |
| LG08_3 | 3,960,000 | 4,340,000 | 380,000 |
| LG12_1 | 15,240,000 | 16,180,000 | 940,000 |
| LG14_1 | 1,100,000 | 1,700,000 | 600,000 |
| LG16_1 | 14,500,000 | 14,740,000 | 240,000 |
| LG18_1 | 21,000,000 | 21,260,000 | 260,000 |
| LG19_1 | 5,020,000 | 5,120,000 | 100,000 |
| LG22_1 | 17,560,000 | 17,680,000 | 120,000 |

Table S5. We identified 68 gene regions that were similar to known genes in other species. These genes, numbered 1- 68, all contained at least 1 SNP from the final dataset (nSNP). *F*_ST_ over all SNPs within each gene region was averaged, mean(*F*st). The linkage group, position, alias, notes, and gene function are provided.

Table S6. Estimated chlorophyll, depth, salinity, temperature, and current velocity, for each sampling location and year, 2003-2017.

Table S7. Pearson’s correlation coefficient calculated between pairwise comparisons of Bayes Factor (BF) and *XTX*_st_ of the three runs done for BayPass for pools in the EBS-WA superpool comparisons, and pools in the EBS-AI superpool comparisons. All correlations were significant.

| Comparison | Salinity | Velocity | Chlorophyll | Temperature | Statistic | Case |
| --- | --- | --- | --- | --- | --- | --- |
| 1 vs. 2 | 0.5049 | 0.5227 | 0.2554 | 0.4354 | BF | EBS-AI |
| 1 vs. 3 | 0.5045 | 0.5236 | 0.2546 | 0.4321 | BF | EBS-AI |
| 2 vs. 3 | 0.5035 | 0.5231 | 0.2556 | 0.4341 | BF | EBS-AI |
| 1 vs. 2 | 0.4072 | 0.3939 | 0.5625 | 0.4435 | *XTX*_st_ | EBS-AI |
| 1 vs. 3 | 0.4045 | 0.3958 | 0.5626 | 0.4433 | *XTX*_st_ | EBS-AI |
| 2 vs. 3 | 0.4059 | 0.3970 | 0.5635 | 0.4418 | *XTX*_st_ | EBS-AI |
| 1 vs. 2 | 0.3372 | 0.3057 | 0.2420 | 0.3916 | BF | EBS-WA |
| 1 vs. 3 | 0.3376 | 0.3036 | 0.2433 | 0.3919 | BF | EBS-WA |
| 2 vs. 3 | 0.3379 | 0.3035 | 0.2433 | 0.3925 | BF | EBS-WA |
| 1 vs. 2 | 0.3457 | 0.3961 | 0.4125 | 0.3244 | *XTX*_st_ | EBS-WA |
| 1 vs. 3 | 0.3439 | 0.3954 | 0.4095 | 0.3267 | *XTX*_st_ | EBS-WA |
| 2 vs. 3 | 0.3442 | 0.3952 | 0.4116 | 0.3251 | *XTX*_st_ | EBS-WA |

Table S8. GATK quality filters for nuclear and mitochondrial data. Nuclear refers to linkage group and scaffold alignments. QD (QualbyDepth) refers to the variant confidence divided by the unfiltered depth of the non-homozygous reference genotypes. FS is the phred-scaled probability that there is strand bias at the site. MQ (RMS Mapping Quality) is the root mean square mapping quality over all the reads at the site. MQRankSum refers to the Z-score from a Wilcoxon rank sum test of the alternate vs the reference read mapping qualities. ReadPosRankSum is the Z-score from a Wilcoxon rank sum test of the alternate vs the reference read position bias. SOR is the strand bias estimated by the symmetric odds ratio test. DP is the approximate read depth (reads with MQ=255 or with bad mates are not included).

| Gene region |  |
| --- | --- |
| nuclear and mitochondrial | --filter-expression "QD < 2.0" |
| nuclear and mitochondrial | --filter-expression "FS > 60.0" |
| nuclear and mitochondrial | --filter-expression "MQ < 55.0" |
| nuclear and mitochondrial | --filter-expression "MQRankSum < -12.5" |
| nuclear and mitochondrial | --filter-expression "ReadPosRankSum < -8.0" |
| nuclear and mitochondrial | --filter-expression "SOR > 3.0" |
| nuclear and mitochondrial | --genotype-filter-expression "DP < 2.0" |
| nuclear and mitochondrial | --disable-read-filter NotDuplicateReadFilter |
| nuclear only | --filter-expression "DP > 6214.0" |
| mitochondrial only | --filter-expression "DP > 32754.0" |
